# Rewiring mitotic checkpoint control *via* Mps1 inhibition overcomes DNA repair-mediated resistance in glioblastoma

**DOI:** 10.1016/j.jbc.2026.113487

**Published:** 2026-08-25

**Authors:** Gaurav Rai, Sivapriya Kirubakaran

**Affiliations:** Department of Chemistry, Indian Institute of Technology Gandhinagar, Gandhinagar, Gujarat, India

**Keywords:** glioblastoma multiforme, Mps1 inhibitor, spindle assembly checkpoint, DNA damage response, kinase inhibitor

## Abstract

Glioblastoma multiforme (GBM) is the most common and aggressive form of primary brain cancer in adults, and treatment is frequently limited by tumor resistance to temozolomide (TMZ), the standard-of-care chemotherapy. This resistance is often driven by the tumor cell's enhanced capacity to repair TMZ-induced DNA damage. Cell division is normally controlled by two quality-control systems: the spindle assembly checkpoint (SAC), which ensures accurate chromosome segregation during mitosis, and the DNA damage response, which detects and repairs genomic damage. Growing evidence suggests these two systems are functionally connected, but whether this connection can be exploited pharmacologically in cancer remains unclear. Here, we redesigned a brain-penetrant chemical scaffold to develop G17, a small molecule that selectively inhibits monopolar spindle 1 (Mps1), the central kinase controlling SAC signaling. Characterization of G17 in biochemical and cellular models showed that Mps1 inhibition forces GBM cells to exit mitosis prematurely, resulting in persistent DNA damage and impaired long-term tumor cell growth. Notably, G17 remained active in TMZ-resistant glioblastoma cells that express O^*6*^-methylguanine-DNA methyltransferase (MGMT), the enzyme primarily responsible for TMZ resistance, indicating that its activity does not depend on MGMT-mediated DNA repair. Together, these findings provide pharmacological evidence that disrupting SAC signaling can expose a DNA repair vulnerability in glioblastoma and identify Mps1 inhibition as a candidate strategy warranting further investigation in treatment-resistant disease.

Cell cycle checkpoints safeguard genomic integrity through regulatory mechanisms that coordinate chromosomal segregation and DNA repair processes. Among these, the spindle assembly checkpoint (SAC) ensures mitotic fidelity during mitosis by delaying anaphase onset until all kinetochores are properly attached to spindle microtubules (1, 2). This checkpoint is mediated by formation of the mitotic checkpoint complex (MCC), which inhibits anaphase-promoting complex/cyclosome (APC/C) and thereby prevents premature mitotic exit (3, 4, 5, 6). A central regulator of SAC signaling is monopolar spindle 1 (Mps1), a dual-specificity kinase that orchestrates kinetochore checkpoint signaling and promotes error correction during chromosome alignment (7, 8). In parallel, cells respond to genotoxic stress through the DNA damage response (DDR), a signaling network that detects DNA lesions, and halts the cell cycle to enable repair or induce apoptosis (9). The DDR comprises a coordinated network of proteins centered on the conserved kinases ATM, ATR, and CHK1 (10). Extensive studies on these master checkpoint regulators have defined the DDR architecture and established its central role in coupling DNA damage sensing to repair, thereby maintaining genomic integrity (10).

Although these pathways are canonically defined by their responses to distinct forms of cellular stress, emerging evidence indicates that their functional boundaries are less discrete than previously presumed, with significant overlapping and crosstalk between them. For instance, several DDR regulators including ATM, BRCA1, RAD9, and ATR have been implicated in enforcing metaphase delay upon microtubule perturbation in both yeast and mammalian settings (11, 12, 13, 14). Consistently, DNA damage can induce a metaphase arrest that is either driven by SAC alone (15, 16, 17, 18, 19) or through combined SAC-DDR signaling (20, 21, 22). Supporting this broader interface, high-throughput studies have identified numerous ATR/ATM substrates among SAC and spindle associated proteins, including MAD1, BUB1, CENPF, NUMA, and NUSAP1 (23, 24). Moreover, recent studies suggest that Mps1 contributes to genome stability pathways by modulating cellular tolerance to genotoxic stress (25, 26, 27, 28, 29). Together, these observations highlight that mitotic checkpoint signaling may influence how tumor cells tolerate DNA damage during proliferation, as sustained SAC activity could extend mitosis and potentially provide additional time for repair of unresolved DNA lesions.

The interplay between checkpoint-controlled mitotic timing and DNA damage tolerance is particularly relevant in glioblastoma multiforme (GBM), an aggressive brain tumor characterized by profound genomic instability (30, 31). The current clinical management for GBM involves surgical tumor excision, radiation-therapy and temozolomide (TMZ)-based chemotherapy. The alkylating agent TMZ remains the standard-of-care for GBM and exert its cytotoxicity by generating DNA lesions, including O^*6*^-methylguanine adducts. However, resistance to DNA-damaging chemotherapy remains a major clinical challenge (32, 33), largely due to the presence of repair mechanisms such as O^*6*^-methylguanine-DNA methyltransferase (MGMT) and alternative genome-stability pathways, thereby limiting therapeutic efficacy (34, 35, 36, 37). Consequently, long-term survival remains poor, with only ∼5% of patients surviving beyond 5 years (33). Notably, elevated expression of SAC component such as Mps1 has been associated with poor patient prognosis in GBM (38, 39, 40, 41, 42, 43, 44, 45), suggesting that mitotic checkpoint signaling may enable tumor cells to tolerate chemotherapy-induced DNA damage. Given the established role of Mps1 in SAC regulation and genome maintenance, we aimed to develop novel Mps1 inhibitors and investigate whether pharmacological inhibition of Mps1 could override mitotic control and promote DNA damage accumulation in glioblastoma. We employed scaffold reprogramming strategy to identify phenothiazine-derived Mps1 inhibitors (PMIs) toward glioblastoma as outlined in Figure 1.

**Figure 1:**
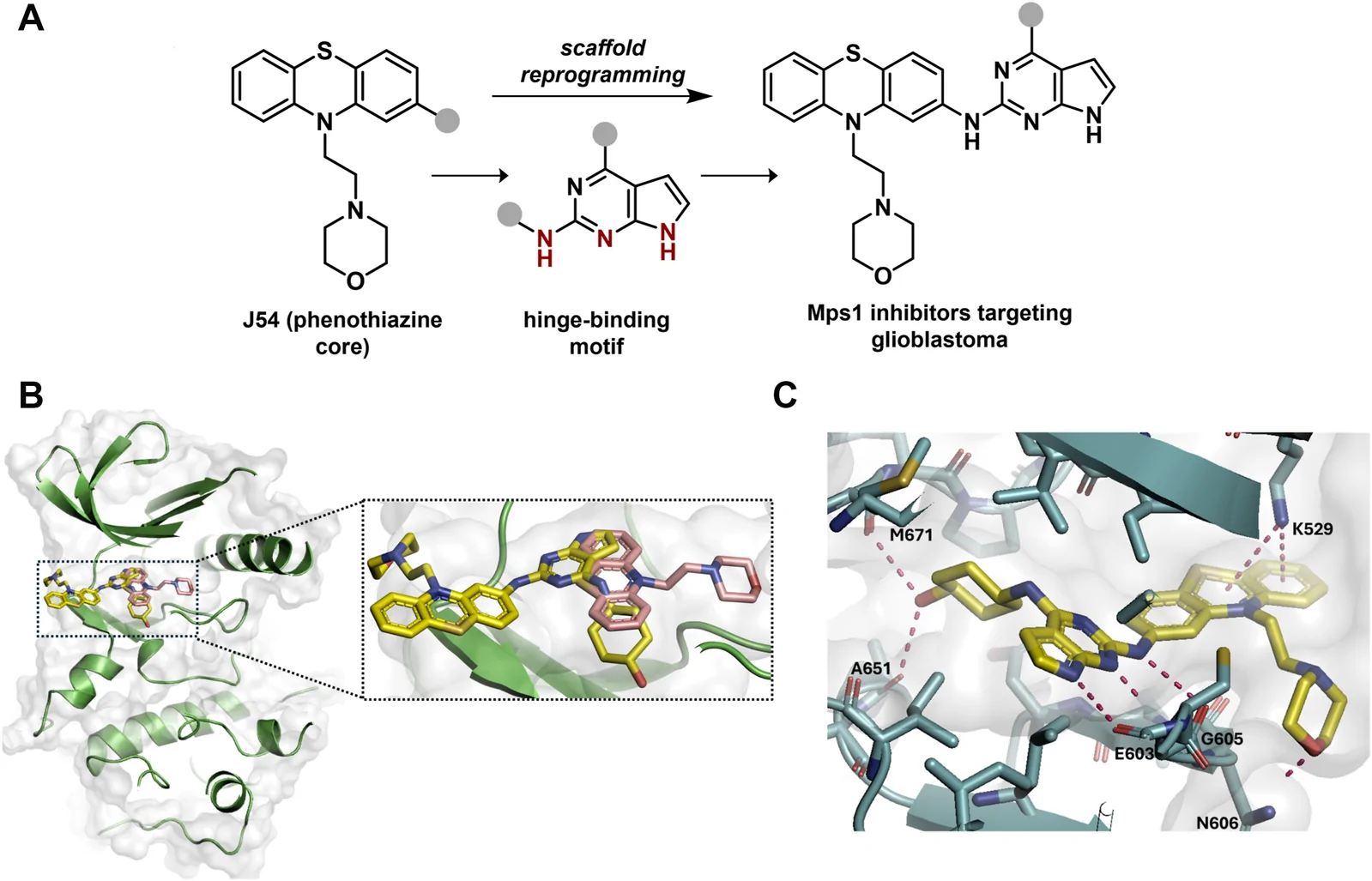
**Scaffold designing of Mps1 inhibitors toward glioblastoma.***A*, scaffold reprogramming strategy used to generate PMIs. The *gray circles* in panel (*A*) denote the point of attachment of different ring/group in the chemical structures (Table 1 and S1). The phenothiazine core was combined with a kinase hinge-binding motif to generate Mps1-targeted analogues. *B*, docking model showing the predicted binding pose of G17 (*yellow*) and J54 (*pink*) within the hinge region of Mps1 (PDB ID: 6B4W). *C*, 3D binding interactions of the designed inhibitor G17 in the binding pocket of Mps1 cocrystal structure (PDB ID 6B4W). The binding site residues are shown within 5 Å. G17 interacts with K529, N606, M671, and A651 while maintaining crucial interactions with E603 and G605, the catalytic domain residues. Representative 2D and 3D modeling images are included in Figure S2. Mps1, monopolar spindle 1; PDB, Protein Data Bank; PMI, phenothiazine-derived Mps1 inhibitor.

## Results

### Scaffold reprogramming-guided design and synthesis of PMIs for glioblastoma

To date, several Mps1 inhibitors have been reported for indications other than GBM (Fig. S1) (46, 47, 48, 49). For example, BAY 1217389 and CFI-402257 (in combination with fulvestrant) have progressed to phase I/II clinical trials for triple-negative breast cancer, whereas CFI-402257 as a monotherapy has been evaluated in a phase I clinical trial for advanced solid tumors. However, none of these agents has achieved clinical approval to date, and their therapeutic potential in glioblastoma remains largely unexplored. Among these, Mps1-IN-3 when combined with vincristine, a microtubule inhibitor, demonstrated only modest preclinical efficacy in an *in vivo* model (38), likely due to inadequate brain exposure. Similarly, another potent Mps1 inhibitor, NTRC 0066-0 exhibited strong *in vitro* activity, yet failed in an *in vivo* GBM models (50), underscoring the challenges of achieving sufficient therapeutic exposure within the brain (51, 52). Together, these examples indicate that achieving adequate brain exposure, rather than target potency alone, has been the primary barrier to advancing Mps1 inhibitors in glioblastoma. Hence, we combined phenothiazine core with a kinase hinge-binding motif to generate Mps1 inhibitors targeting glioblastoma (Fig. 1*A*). The phenothiazine scaffold from our previous report of J54 (53, 54, 55), was selected for its reported ability to cross blood–brain barrier, whereas additional pharmacophoric features were introduced at the C2 position to improve kinase selectivity. Given that purine scaffolds are well-established Mps1 inhibitors (38, 56, 57, 58), we investigated 7*H*-pyrrolo[2,3-d]pyrimidine isostere, that preserves hinge-binding capacity with reduced off-target effects relative to the purine scaffold (59, 60, 61, 62, 63, 64, 65). This strategy enabled identification of a new class of PMIs. Molecular modeling of J54 with an in-house designed Mps1 inhibitor (for *e.g.* G17) revealed that both compounds occupy distinct subregions of the Mps1 active site (Protein Data Bank [PDB] ID: 6B4W, Fig. 1*B*). J54 fits deeply within the ATP cleft, with its ethyl-morpholine pointing inwards but lacking stabilizing polar interactions. In contrast, incorporation of the 7*H*-pyrrolo[2,3-d]pyrimidine moiety in G17 reorients the ethyl-morpholine towards the solvent exposed site, forming an oxygen-mediated interaction with N606, whereas secondary-amine engages G605 (Fig. 1*C*), consistent with canonical hinge binding (Fig. S2).

### G17 selectively inhibits Mps1 and disrupts SAC signaling in glioblastoma

We undertook structural-activity relationship (SAR) exploration by varying R_1_-substituents at C4 position of fused system (Table S1) and synthesized the analogs following optimized protocols (Scheme S1). Biochemical potency was assessed using ADP Glo enzyme assay with full-length Mps1. Compounds were initially screened at 10 and 1 μM, with reversine, MPI-0479605, and staurosporine included as positive controls. In parallel, short-term cytotoxicity was evaluated across LN229, LN18, and U87MG cells exhibiting both MGMT-deficient and -proficient backgrounds (66). All analogs were designed to maintain acceptable BBB permeability. The first series (G13–G16a) bearing metasubstituted aryl systems exhibited strong Mps1 inhibition at 10 μM but reduced potency at 1 μM. Nevertheless, most analogs reduced cell viability across GBM cell models with IC_50_ < 10 μM, outperforming controls G4, J54, and TMZ. Notably, the carboxylic acid analog G15 was inactive up to 20 μM, whereas its prodrug restored activity as expected (Table 1). In second series (G17–G23), the aryl unit was replaced with aliphatic heterocycles, resulting in four of seven derivatives exhibiting >60% Mps1 inhibition at 1 μM. Methyl substitution on piperazine (G18 to G19) impaired Mps1 potency, but enhanced cytotoxicity. Replacing nitrogen with carbon (G19 to G20) reduced cytotoxicity whereas oxygen analogs (G21 and G22) did not provide notable improvement in antiproliferative effects. Among all, trans-amino cyclohexanol analog G17 (Fig. 2*A*) displayed most favorable profile, combining strong Mps1 inhibition at both concentrations with acceptable BBB permeability.

**Table 1:** Summary of biochemical inhibition of Mps1 and cell viability (IC_50_) of PMI analogs


Compound ID	Analogs (R_1_)	Enzyme assay		Cell viability IC_50_ (μM)		
		% Mps1 inhibition (10 μM)^*a*^	% Mps1 inhibition (1 μM)^*b*^	LN229^*c*^	LN18^*d*^	U87MG^*d*^
G4	-	58.6 ± 22.0	<1.0	>10.0	>10.0	>10.0
G13		74.4 ± 0.2	<1.0	4.9 ± 0.15	5.3 ± 0.89	>10.0
G14		90.6 ± 4.0	26.1 ± 3.7	3.7 ± 1.15	3.8 ± 0.28	7.0 ± 1.02
G15		80.8 ± 17.5	34.2 ± 4.5	>10.0	>10.0	>10.0
G16		97.2 ± 6.5	30.7 ± 3.0	4.8 ± 1.22	2.7 ± 0.55	4.6 ± 1.62
G16a		98.6 ± 9.2	40.6 ± 2.0	>10.0	>10.0	>10.0
G17		>99.0	>99.0	0.6 ± 0.10	0.8 ± 0.09	1.1 ± 0.78
G18		>99.0	63.9 ± 2.6	4.4 ± 0.26	7.7 ± 1.45	5.6 ± 0.38
G19		>99.0	39.2 ± 2.8	2.5 ± 1.44	3.0 ± 0.54	6.3 ± 0.08
G20		90.0 ± 8.3	42.8 ± 3.4	6.2 ± 0.98	6.5 ± 0.49	6.0 ± 1.23
G21		86.2 ± 3.8	67.9 ± 2.3	5.4 ± 0.76	4.1 ± 0.66	4.1 ± 1.10
G22		47.0 ± 6.2	42.6 ± 2.3	4.9 ± 1.42	5.1 ± 0.62	nd
G23		72.7 ± 5.7	70.4 ± 2.4	2.6 ± 0.21	4.5 ± 0.89	4.8 ± 0.78
J54^*e*^	-	>99.0	38.7 ± 2.8	48.7	37.8	52.3
Reversine	-	>99.0	92.1 ± 1.2	-	-	-
MPI-0479605	-	>99.0	98.7 ± 1.3	-	-	-
Staurosporine	-	>99.0	83.2 ± 0.3	-	-	-
TMZ^*e*^	-	-	-	105.9	nd	93.6

^*a,b*^ % Mps1 inhibition determined at 10 μM and 1 μM, respectively. The enzyme inhibition values represent mean ± SD of two independent experiments (n = 2).

^*c,d*^ Cytotoxicity values representing mean ± SD of three and two independent experiments, respectively (n = 3 for *c*, and n = 2 for *d*).

^*e*^ IC_50_ value of controls (J54 and TMZ) is reported as per the reference no. 55. See also Table S1.

Mps1, monopolar spindle 1; PMI, phenothiazine-derived Mps1 inhibitor; nd, not determined.

**Figure 2:**
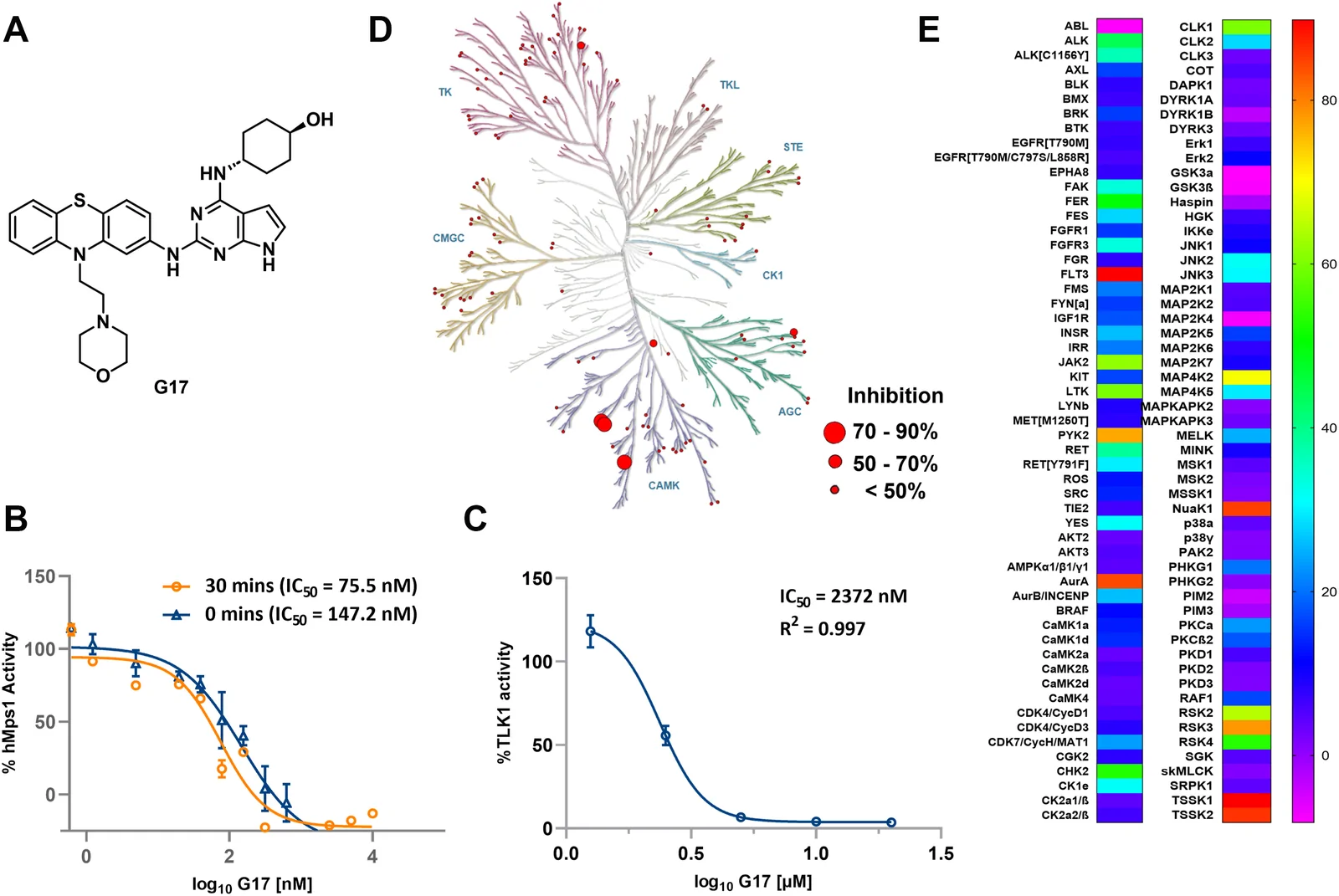
**G17 selectively inhibits full-length Mps1 in an*****in vitro*****enzyme assay.***A*, chemical structure of G17. *B*, dose–response curve of G17 against full-length Mps1 determined using an ADP-Glo kinase assay. IC_50_ values represent mean ± SD from two independent experiments (n = 2). *C*, dose–response curve of G17 against recombinantly purified full-length TLK1 determined using an ADP-Glo kinase assay. IC_50_ values represent mean ± SD from two independent experiments (n = 2). *D*, kinome profiling dendrogram with (*E*) heatmap of G17 at 1 μM across a curated kinome panel and % inhibition values are summarized in Table S2. The dendrogram in panel (*D*) was sourced from kinhub.org. Mps1, monopolar spindle 1; TLK1, Tousled-like kinase 1.

Dose–response analysis confirmed G17-mediated potent inhibition of full-length Mps1 with an IC_50_ of 75.5 nM (Fig. 2*B*), and >30-fold selectivity over Tousled-like kinase 1 (TLK1) (IC_50_ = 2372 nM, Fig. 2*C*), indicating preliminary kinase selectivity. Kinome profiling of G17 at 1 μM against a curated panel of kinases further revealed a favorable selectivity profile, with no kinase inhibited by more than 90% (Fig. 2, *D* and *E* and Table S2) and only weak inhibition of Aurora B and CDK4/7 (<20%). Given the previous reports of Mps1 overexpression in GBM, we first examined its basal level across available GBM cell models. Notably, Mps1 expression was significantly elevated in TMZ-resistant GBM tumors (hereafter referred to as LN229-TMZ-R) (54) compared to TMZ-sensitive cells (Fig. 3*A*), suggesting its potential role in acquired-drug resistance. We next assessed cellular Mps1 inhibition in both TMZ-sensitive and TMZ-resistant LN229 cells. Target engagement was evaluated by immunoblotting for phosphorylation of Mps1 at T676 (67) following G17 treatment, which resulted in a substantial reduction in phospho-Mps1 levels (Fig. 3, *C*–*E*), indicative of SAC disruption. In nocodazole-arrested cells, G17 treatment also decreased phospho-histone H3 levels, a widely used surrogate marker of Mps1 inhibition (68, 69) (Fig. 3, *F*–*H*), thereby alleviating G2/M arrest and promoting mitotic exit. Collectively, these results demonstrate that G17 selectively inhibits Mps1 and compromises SAC signaling in both drug-sensitive and drug-resistant GBM cells.

**Figure 3:**
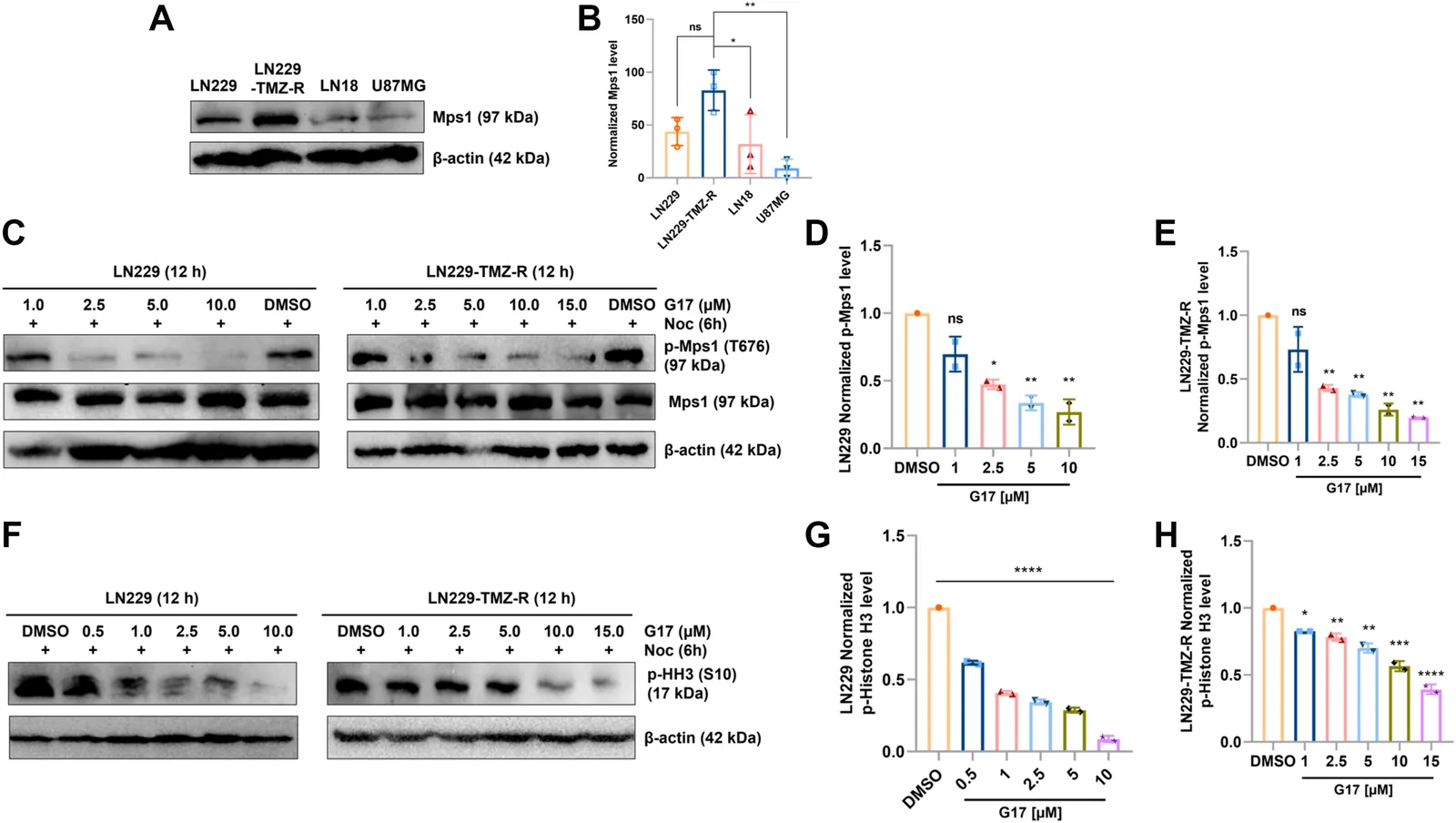
**Cellular Mps1 inhibition in GBM cells.***A*, basal expression profile of Mps1 across available glioblastoma cells immunoblotted against anti-Mps1 antibody. *B*, represents quantification of Mps1 levels from panel (*A*), and statistical significance was determined by one-way ANOVA, ns = not significant, ∗*p* < 0.05, and ∗∗*p* < 0.01 compared to LN229-TMZ-R group. *C*, western blot analysis showing suppression of phospho-Mps1 (T676) following G17 treatment in TMZ-sensitive LN229 and TMZ-resistant LN229-TMZ-R cells. Cells were synchronized with nocodazole (100 ng/mL, 6 h) prior to treatment. (*D*) and (*E*) represents quantification of p-Mps1 levels from panel (*C*) and statistical significance was determined by one-way ANOVA, ns = not significant, ∗*p* < 0.05, and ∗∗*p* < 0.01 compared to DMSO group. *F*, reduction of phospho-histone H3 (Ser10) levels following G17 treatment in nocodazole-arrested cells, indicating disruption of SAC signaling and premature mitotic progression. (*G*) and (*H*) represents quantification of p-HH3 levels from panel (*F*) and statistical significance was determined by one-way ANOVA, ∗*p* < 0.05, ∗∗*p* < 0.01, ∗∗∗*p* < 0.001, and ∗∗∗∗*p* < 0.0001 compared to LN229-TMZ-R group. DMSO, dimethyl sulfoxide; Mps1, monopolar spindle 1; SAC, spindle assembly checkpoint; TMZ, temozolomide.

### Mps1 inhibition promotes DNA damage accumulation and impair repair kinetics in glioblastoma

Several chemotherapeutic agents target genomic DNA and induce distinct spectra of DNA lesions. Cisplatin forms intrastrand and interstrand DNA crosslinks (ICLs) that can be processed into DNA strand breaks during replication and repair (70), bleomycin generates single- and double-strand breaks through iron-dependent free-radical attack on the deoxyribose backbone (71), and TMZ produces O^6^-methylguanine adducts that trigger futile cycles of mismatch repair, ultimately resulting in DNA double-strand breaks (72). Because SAC signaling can prolong mitosis and permit resolution of DNA lesions, and since Mps1 participates in genome-stability pathways, we investigated whether pharmacological inhibition of Mps1 promotes DNA damage accumulation and disrupt repair kinetics in glioblastoma. DNA damage was quantified by monitoring phosphorylation of histone H2AX (γ-H2AX), a surrogate marker of DNA double-strand break (DSB) formation and several non-DSB lesions (Fig. 4) (73, 74). In LN229 cells, cotreatment with G17 and TMZ resulted in ≥2.5-fold increase in γ-H2AX intensity relative to either agent alone (Fig. 4, *A* and *C*). A similar trend was observed in LN229-TMZ-R cells, where combined treatment resulted in an approximately two-fold increase in γ-H2AX levels, whereas TMZ alone generated minimal signal even at 200 μM (Fig. 4, *B* and *D*), consistent with the resistant phenotype.

**Figure 4:**
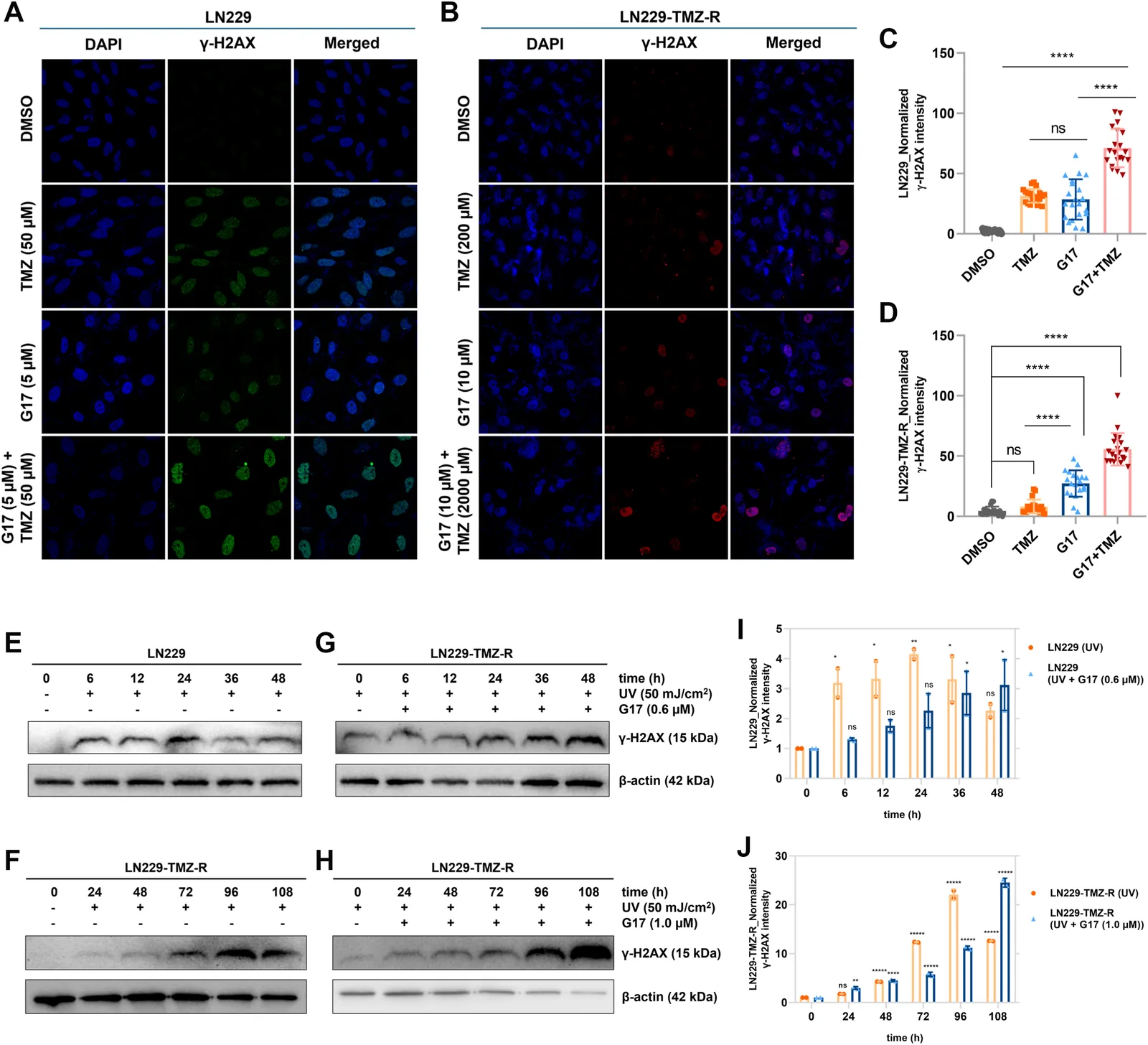
**G17-mediated Mps1 inhibition induces persistent DNA damage and delays repair kinetics.** Representative immunofluorescence images showing γ-H2AX accumulation in different pseudocolour channels (*A*) LN229 (*green*) and (*B*) LN229-TMZ-R (*red*) cells following treatment with control (0.1% DMSO), TMZ (50 μM for LN229 cells and 200 μM for LN229-TMZ-R cells), or TMZ + G17 for 48 h. (*C*) and (*D*) are quantification of γ-H2AX fluorescence intensity from panels (*A*) and (*B*), respectively, calculated from 20 individual cells. Statistical significance was determined by one-way ANOVA, ns = not significant and ∗∗∗∗*p* < 0.0001 compared to DMSO group. Further immunofluorescence analyses were shown in Figure S3. Representative immunoblots showing γ-H2AX dynamics following UV-induced DNA damage (50 mJ/cm^2^) in absence of G17 in (*E*) LN229 and (*F*) LN229-TMZ-R cells. (*G*) and (*H*) represents immunoblot analyses of γ-H2AX persistence (delayed DNA damage resolution) in the presence of G17. *I*, represents quantification of γ-H2AX levels from panels (*E*) and (*G*), respectively. Statistical significance was determined by one-way ANOVA, ns = not significant, ∗∗*p* < 0.01, ∗∗∗*p* < 0.001, and ∗∗∗∗*p* < 0.0001 compared to control group. *J*, represents quantification of γ-H2AX levels from panels (*F*) and (*H*), respectively. Statistical significance was determined by one-way ANOVA, ns = not significant, ∗∗*p* < 0.01, ∗∗∗*p* < 0.001, and ∗∗∗∗*p* < 0.0001 compared to control group. DMSO, dimethyl sulfoxide; Mps1, monopolar spindle 1; TMZ, temozolomide.

Mps1-mediated phosphorylation of CHK2, a key DNA repair protein, at T68 promotes its activation (25, 26), leading to downstream phosphorylation of p53 and Cdc25. Moreover, inhibition of Mps1 has been shown to impair both homologous-directed repair (HDR) and non-homologous end joining (NHEJ), two primary DNA DSB repair pathways (42, 75). We therefore hypothesized that pharmacological Mps1 blockade using G17 would result in the sustained DNA damage that would otherwise be resolved through HDR or NHEJ. To evaluate DNA repair kinetics, cells were subjected to UV-induced DNA damage (50 mJ/cm^2^) in the presence or absence of G17, and γ-H2AX levels were monitored over time. In the absence of G17, UV-induced γ-H2AX signal peaked at 24 h in LN229 cells (Fig. 4, *E* and *I*) and at 96 h in LN229-TMZ-R cells (Fig. 4, *F* and *J*), followed by a decline at the subsequent time point in each series. In contrast, cotreatment with G17 markedly prolonged γ-H2AX persistence with signal continued to increase without decline through 48 h in LN229 cells (Fig. 4, *G* and *I*) and through 108 h in LN229-TMZ-R cells (Fig. 4, *H* and *J*). These results indicate that pharmacological inhibition of Mps1 delays DNA damage resolution, supporting a role for SAC signaling in coordinating mitotic progression with genome repair processes in GBM cells.

### Mps1 inhibition triggers apoptotic cell death in glioblastoma cells

To determine whether G17-induced persistent DNA damage compromises the long-term proliferative capacity, we performed clonogenic survival assay, a stringent measure of cellular survival beyond short-term metabolic viability readouts. Notably, G17 markedly suppressed clonogenic potential, resulting up to an 8-fold reductions at 5.0 μM in TMZ-sensitive cells and more pronounced up to 75-fold decrease at 10.0 μM in TMZ-resistant cells (Figs. 5, *A*–*D* and S4), relative to control. The reduction in clonogenic survival paralleled the decrease in short-term viability observed across GBM cell models, including LN229 and U87MG cells lacking MGMT as well as LN18 cells expressing functional MGMT (66). Interestingly, G17 retained activity in LN229-TMZ-R cells, with an IC_50_ of 2.0 μM (Fig. 5*E* and Table 1). The comparable response across GBM models with distinct MGMT status and TMZ sensitivity suggests MGMT-independent activity of G17 and highlights its potential to bypass established routes of TMZ resistance.

**Figure 5:**
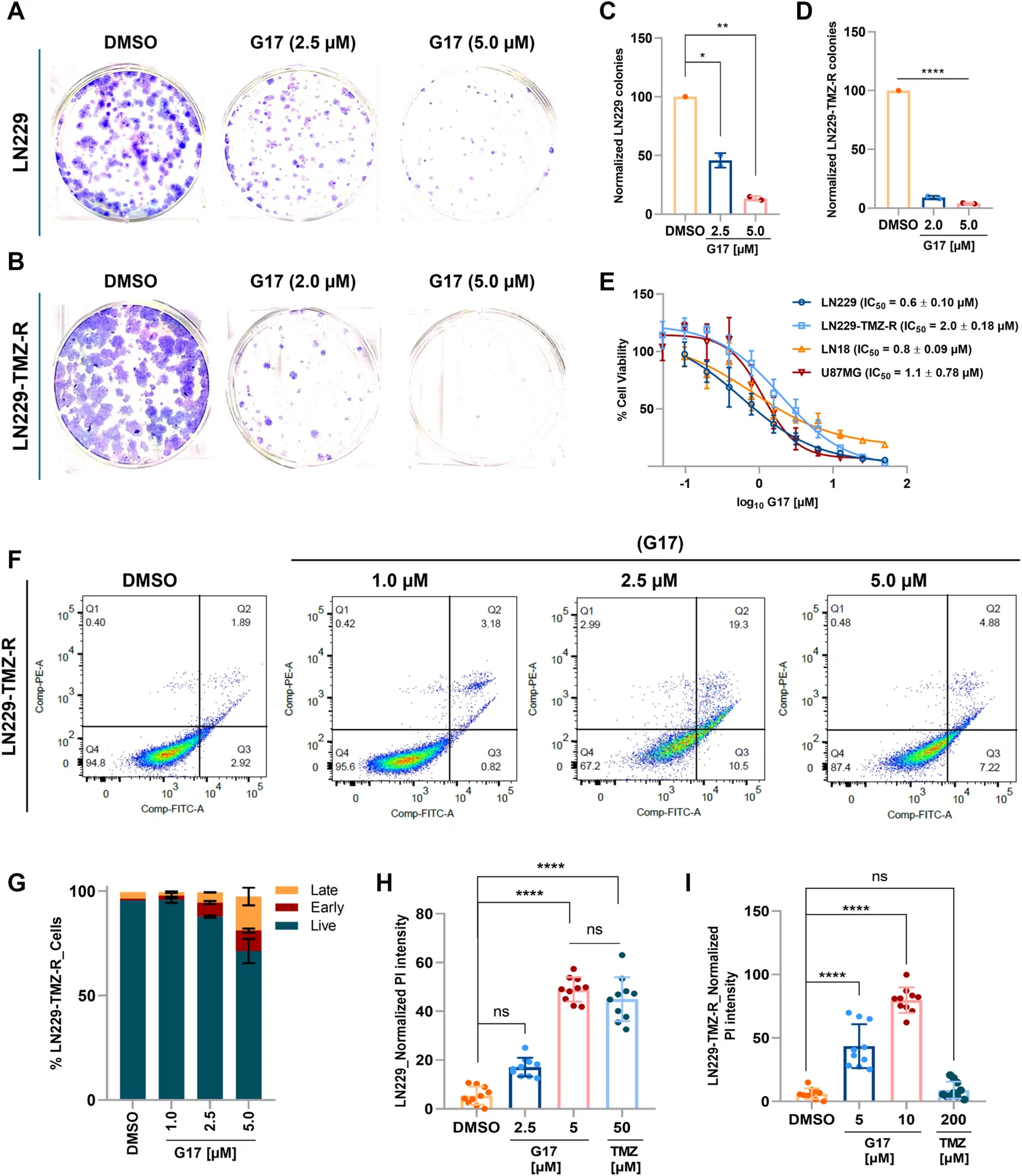
**G17-induced Mps1 inhibition leads to loss of clonogenic survival and apoptotic cell death in glioblastoma cells.** Representative images of cell colonies formed by (*A*) LN229 and (*B*) LN229-TMZ-R cells following treatment with G17. *C* and *D*, quantification of colony formation from panels (*A*) and (*B*), respectively. Statistical significance was determined by one-way ANOVA, ns = not significant, ∗*p* < 0.05, ∗∗*p* < 0.01, and ∗∗∗∗*p* < 0.0001 compared to DMSO group. Further clonogenic images and quantification were shown in Figure S4. (*E*) Dose-dependent analysis of G17 representing short-term cytotoxicity across all GBM cells (LN229, LN229-TMZ-R, LN18, and U87MG). IC_50_ values represent mean ± SD (n = 4). *F*, apoptosis analysis using annexin-V/PI staining in GBM cells post treatment with G17. *G*, represents quantification of early (Annexin V-positive/PI-negative) and late (Annexin V-positive/PI-positive) apoptotic cells from panel (*F*). (*H*) and (*I*) are quantification of PI/Hoechst staining cell death assay in LN229 and LN229-TMZ-R cells, respectively. Statistical significance was determined by one-way ANOVA, ns = not significant and ∗∗∗∗*p* < 0.0001 compared to DMSO group. Representative cell death images are shown in Figure S5. DMSO, dimethyl sulfoxide; Mps1, monopolar spindle 1; PI, propidium iodide; TMZ, temozolomide.

Given that Mps1 inhibition impaired DNA repair kinetics, we hypothesized that persistent DNA lesions would ultimately trigger apoptotic cell death in GBM. Apoptosis was therefore assessed using dual Annexin V/propidium iodide (PI) staining. In LN229-TMZ-R cells, G17 treatment (5 μM) increased early and late apoptotic cell populations by ∼3.5-fold and ∼10-fold, respectively, compared with vehicle control (Fig. 5, *F* and *G*). We next quantified GBM cell death using PI/Hoechst staining in both TMZ-sensitive and TMZ-resistant cells. PI selectively labels membrane-compromised (dead) cells whereas Hoechst stains total nuclei. Consistent with above findings, PI/Hoechst staining revealed a significant increase in GBM cell death following G17 treatment in both TMZ-sensitive and TMZ-resistant models. In LN229 TMZ-sensitive cells, G17 induced cell death comparable to TMZ at ∼10-fold lower concentration (Fig. 5H and S5), whereas in -resistant cells it produced ∼8-fold greater cell death than TMZ (Fig. 5*I*). Collectively, these findings demonstrate that pharmacological disruption of SAC signaling by G17 promotes persistent DNA damage, leading to loss of clonogenic potential and apoptotic collapse of glioblastoma cells irrespective of TMZ resistance status. These results further support the therapeutic potential of SAC perturbation as a viable strategy to exploit genomic vulnerabilities in glioblastoma.

## Discussion

Our findings support a functional link between SAC and DDR signaling in glioblastoma. Pharmacological inhibition of Mps1, a key SAC regulator, promoted premature mitotic exit and was accompanied by persistent DNA damage, as reflected by sustained γ-H2AX signaling and delayed damage resolution. These observations are consistent with prior reports linking Mps1 to genome stability and DNA damage signaling (25, 26, 28, 29) and extend these findings to a pharmacological setting in glioblastoma, including TMZ-resistant cell models.

To generate a brain-penetrant Mps1 inhibitor, we incorporated a hinge-binding heterocycle into a phenothiazine scaffold, a chemotype previously associated with blood–brain barrier permeability. This design allowed G17 to engage Mps1 whereas retaining selectivity across the kinase panel tested. Several previously reported Mps1 inhibitors have shown potent biochemical and cellular activity and advanced to clinical evaluation in non-CNS malignancies (76, 77, 78, 79); their application in glioblastoma has been more limited (38, 50), in part because CNS efficacy requires adequate brain exposure in addition to target potency. The phenothiazine-derived scaffold used here represents one possible starting point for further optimization of brain-penetrant Mps1 inhibitors, though this will require direct pharmacokinetic evaluation.

GBM treatment resistance remains a substantial clinical problem, and efficacy of standard-of-care, TMZ, is frequently limited by MGMT-mediated repair and other DNA damage tolerance mechanisms (80, 81, 82). Approaches that increase DNA damage burden or directly inhibit repair pathways have shown activity but are often constrained by adaptive resistance (83). That G17 retained activity in TMZ-resistant cells suggests that Mps1 inhibition may act through a mechanism not dependent on the same resistance pathways that limit TMZ efficacy, though this remains to be tested directly.

Several aspects of the underlying mechanism remain unresolved. Although sustained γ-H2AX signaling indicates impaired genome maintenance, we did not directly assess the relative contribution of HDR *versus* NHEJ, or the status of specific DDR regulators. Future work will examine the effect of Mps1 inhibition on CHK1, CHK2, BRCA1, and RAD51, and possible crosstalk with TGF-β/SMAD signaling, to clarify the basis of the observed repair defect. Pharmacokinetic and *in vivo* studies will also be performed to evaluate brain exposure, efficacy, and safety of G17 in glioblastoma models before its therapeutic potential can be more fully established.

## Experimental procedures

### Cell culture

The human glioblastoma cell lines LN229, LN18, and U87MG were obtained from the National Cell Repository at NCCS, Pune, India. The TMZ-resistant LN229-TMZ-R cell line was generated as previously reported (54) through intermittent treatment of LN229 cells with gradually increasing concentrations of TMZ (50–200 μM), interspersed with recovery periods in drug-free medium. All cell lines were cultured in Dulbecco’s Modified Eagle Medium (DMEM) supplemented with 10% fetal bovine serum (Gibco) and 1% penicillin–streptomycin. Cells were maintained at 37 °C in a humidified incubator containing 5% CO_2_.

### hMps1 biochemical enzyme assay

For enzyme inhibition assay, full-length recombinant Mps1 (1–857 amino acid residue) expressed as GST-fusion protein (N-terminal) from Carna Biosciences (Cat# 05-169) was used. Mps1-mediated phosphorylation of MBP-peptide substrate (APRTPGGRR, Medchem Express, Cat# HY-P2456) was monitored using ADP-Glo kinase assay kit (Promega Corp, Cat# V6930). All the test compounds were prepared in ADP-Glo kinase assay buffer (40 mM Tris–Cl, pH 7.4, 20 mM MgCl_2_, 0.1 mg/ml bovine serum albumin, and 5 mM DTT in milli-Q water). In a reaction well, active Mps1 (20 ng final assay concentration in 15 μl) was incubated with test compounds (<0.1% dimethyl sulfoxide [DMSO]) for 0 or 30 min at 25 °C, and the reaction was immediately started by adding pure ATP (100 μM final assay concentration) and substrate (1 μM final assay concentration) mixture for 1 h and then stopped by adding ADP-Glo reagent for 30 min. Following this, a kinase detection reagent was added and incubated for 1 h in a dark room. Luminescence was recorded using Envision multimode plate reader (PerkinElmer, Inc, MA) with an integration time of 1 s per well. Data analysis and curve fitting were performed using GraphPad prism 8.3.0 software. The experiments were performed in duplicate.

The expression and purification of human-TLK1 was done as per the previously reported procedure from Bhoir *et al.* (84) Briefly, full-length human TLK1 (549 amino acids; UniProt Q9UKI8-3) was cloned into the pETDUET-1 vector with an N-terminal 6xHis tag and coexpressed with bacteriophage λ protein phosphatase in *Escherichia coli* Rosetta-Gami 2 (DE3) pLysS cells to obtain soluble, unphosphorylated protein, as described previously. Cells were grown in LB medium supplemented with chloramphenicol and ampicillin and induced with 0.5 mM IPTG at an O.D._600_ of 0.6 to 0.8, followed by expression at 25 °C for 9 h. Harvested cells were lysed by sonication in Tris-buffer containing NaCl, Triton X-100, glycerol, TCEP, imidazole, and protease inhibitors. After clarification by centrifugation, recombinant 6× His-tagged TLK1B was purified by Ni-NTA affinity chromatography, eluted with imidazole-containing buffer, concentrated using Amicon Ultra centrifugal filters (10 kDa cutoff), and further purified by size-exclusion chromatography on a Superdex 75 column. Protein purity and homogeneity were assessed by SDS-PAGE analysis.

### CellTiter-Glo assay

The assay was performed using the CellTiter-Glo ([CTG] Promega Corp., Cat# G7572) reagent as follows: Cells growing in logarithmic phase were trypsinized and counted with a hemacytometer using trypan blue dye. Approximately 2000 cells/well were seeded in 96-well white plates and left to adhere for 24 h. Next day, respective treatments with compounds were administered for 72 h. Following that, 5 μl of CTG reagent in each well were added in the dark and incubated at room temperature for 10 min to allow proper mixing. Furthermore, luminescence was recorded using EnVision multimode plate reader (PerkinElmer, Inc, MA). Readings for the compound treatment groups were normalized with DMSO and IC_50_ of compounds were analyzed using GraphPad Prism.

### Clonogenic survival assay

Approximately 1000 cells were seeded in 6-well plates in individual wells. Post 24 h of seeding, compounds were treated with their respective concentrations and incubated for 72 h. Following that, compound treated media was carefully removed, fresh media were added, and cells were allowed to grow for 10 to 14 days. Subsequently, media were removed, cells were washed with 1× PBS, cell colonies were fixed with 100% methanol (4 °C, 20 min) and stained with 0.5% crystal violet solution for 15 min. Colonies with >50 cells were counted in an inverted microscope, and data were analyzed using GraphPad Prism.

### Apoptosis assay

For apoptosis analysis, the cells were treated with respective concentration of compounds for 72 h. After trypsinization, cells were harvested, centrifuged, and resuspended in 1× Annexin Binding Buffer (Invitrogen, Cat# V13246). Subsequently, Annexin V (Invitrogen, Cat# R37174) and PI (Invitrogen, Cat# F10797) were added as per manufacturer’s protocol and cells were incubated in dark for 20 min before acquisition. Samples were acquired using BD FACSAria Fusion Flow Cytometer, and data were analyzed using FlowJo software.

### Immunoblotting

Before harvesting, both LN229 and LN229-TMZ-R cells were synchronized with nocodazole (100 ng/ml final concentration) for 6 h and given compound treatment for 12 h. Following that, cells were trypsinized, harvested, and washed with 1x PBS twice. Cells were then lysed using optimized lysis buffer [50 mM Tris–Cl (pH 7.5), 250 mM NaCl, 1.0% NP-40, and 5 mM EDTA] supplemented with 1x protease and phosphatase inhibitor cocktail (Cell Signaling Technology, Cat# 5872). Protein concentrations were normalized, and equal amounts of samples were separated using SDS-PAGE. Next, proteins were transferred to the polyvinylidene difluoride membrane (PVDF) (0.22 μm), and membranes were blocked with 5% skimmed milk solution. Subsequently, membranes were washed thrice with TBST and incubated with respective antibodies overnight at 4 °C. Later, membranes were washed and exposed to horseradish peroxidase (HRP) conjugated secondary antibody for 1 to 2 h. Protein bands were visualized using enhanced chemiluminescence (ECL) substrates and imaging was done using Bio-Rad ChemiDoc Go imaging system. Images were analyzed using ImageLab software.

Following antibodies were used for immunoblotting: Mps1 antibody (Cell Signaling Technology, Cat #3255 or Santa Cruz biotechnology, Cat #sc-56968), Phospho-Mps1 (Thr676) Polyclonal Antibody (Invitrogen, Cat #PA5-64614), Phospho-Histone H3 (Ser10) antibody (Cell Signaling Technology, Cat #53348), beta-Actin Antibody (C4) HRP antibody (Santa Cruz Biotechnology, Cat #sc-47778 HRP), Phospho-Histone H2A.X (Ser139) (Cell Signaling Technology, Cat #2577S), Anti-rabbit IgG, HRP-linked Antibody (Cell Signaling Technology, Cat #7074).

### Live-dead assay

1 × 10^3^ cells were seeded in 96-black well plates with transparent bottom surface and left the cells to adhere for 24 h. Following this, compounds were treated with respective concentrations and incubated for 72 h. After incubation, compound treated media was removed and fresh serum containing media with PI (2.5 μg/ml) and Hoechst (2.0 μg/ml) was added and incubated for 15 min in CO_2_ incubator at 37 °C. Fluorescence intensity was measured, quantified, and imaged using confocal laser scanning microscope.

### Immunofluorescence assay

Cells were cultured on 18 mm coverslip on a 12-well plate at a density of 5 × 10^3^ and incubated at 37 °C at 5% CO_2_ for 24 h. Next day, cells were treated with compounds at respective concentrations and incubated for 48 h. After treatment, media were carefully aspirated; cells were washed with 1× PBS, fixed with 2% paraformaldehyde (PFA), and permeabilized with methanol. Next, cells were blocked with a blocking solution (5% fetal bovine serum + 0.3% Triton X-100 in 1× PBS) for 1 h at room temperature and probed with γ-H2AX primary antibody (Cell Signaling Technology, Cat# 2577, 1: 500) overnight at 4 °C, followed by the incubation with the Anti-rabbit IgG Alexa Fluor-488-labeled secondary antibody (Cell Signaling Technology, Cat# 4412, 1:500) for 2 h at room temperature. Cells were mounted with 4′,6-diamidino-2-phenylindole (DAPI) (Abcam, Cat# ab104139), imaged, and quantified with confocal laser scanning microscope and ImageJ software, respectively.

### Kinome profiling assay

Kinome profiling of G17 was carried out at Carna Bioscience, Japan, over a panel of 110 kinases at 1 M concentration. The screening was carried out using either mobility shift assay or immobilized metal ion affinity-based fluorescence polarization assay. Kinases with >20% inhibition on their respective kinase panel from already reported Mps1 inhibitors (53, 61, 62, 63, 64) were chosen as a modified target panel. This reported Mps1 inhibitors from ref. no (53, 61, 62, 63, 64). were chosen due to the presence of the similar pharmacophore moiety (J54 and 7*H*-pyrrolo[2,3-d]pyrimidine).

### Molecular docking studies

Crystal structures of Mps1 (PDB: 6B4W) were retrieved from the PDB and prepared for docking, including hydrogen addition, removal of water molecules, optimization of hydrogen-bonding networks, and energy minimization. For each protein, a receptor grid was centered on the centroid of the cocrystallized ligand within its respective active site, and grid accuracy was validated by redocking the native ligand back into the corresponding binding pocket. Two-dimensional structures of G17 and J54 were generated using a 2D sketcher and processed through ligand preparation without structural constraints, generating all energetically reasonable tautomers, ionization states, and stereoisomers to capture the full range of possible low-energy conformations. Docking was performed in extra-precision (XP) mode using the Glide module of the Schrödinger suite, with ligands treated as flexible and protein structures held rigid. Docking scores were calculated as XP scores and resulting protein–ligand binding poses were examined using the XP Visualizer and Pymol.

## Data availability

All the data are available upon request.

## Supporting information

This article contains supporting information (38, 53, 55, 76, 77, 78, 79).

## Conflict of interest

The authors declare that they have no conflicts of interest with the contents of this article.
