# Supplementary material for: Rewiring mitotic checkpoint control via Mps1 inhibition overcomes DNA repair-mediated resistance in glioblastoma
Source: J Biol Chem. 2026 Aug 25;302(10):113487. doi: 10.1016/j.jbc.2026.113487 (PMC13631577; doi:10.1016/j.jbc.2026.113487)
Supplement: Supplementary file 1 [file mmc1.docx]

**Rewiring mitotic checkpoint control via Mps1 inhibition overcomes DNA repair-mediated resistance in glioblastoma**

**Gaurav Rai,^1^ Sivapriya Kirubakaran^1^***

^1^Department of Chemistry, Indian Institute of Technology Gandhinagar, Gandhinagar-382055, Gujarat, India.

*Corresponding author: [priyak@iitgn.ac.in](mailto:priyak@iitgn.ac.in) (S.K.)

**Supporting Information**

**Table of contents**

**1. Table S1. Summary of biochemical Mps1 enzyme, BBB values (Schrödinger), and anti-proliferative assay……………………………………………………………………………………………S2**

**2. Table S2. Summary of Kinome profiling of G17………………………………………………………S4**

**3. Figure S1. Representative Mps1 kinase inhibitors reported in the literature…………………….S8**

**4. Figure S2. Binding pose overlay of G17 and J54…………………………………………………….S9**

**5. Figure S3. Representative immunocytochemistry images showing γ-H2AX fluorescence intensity………………………………………………………………………………………………………S10**

**6. Figure S4. Representative Images of cell colonies…………………………………………………S11**

**7. Figure S5. Cell death analysis via dual Hoechst/PI-staining assay………………………………S12**

**8. Chemistry experimental methods……………………………………………………………………..S13**

**9. Scheme S1. Synthesis of phenothiazine-based Mps1 inhibitors………………………………..S14**

**10. Detailed experimental procedures for chemistry………………………………………………….S15**

**11. 1H NMR spectrum, 13C NMR spectrum, and HPLC analysis of final compounds……..……..S31**

**12. References……………………………………………………………………………………………….S57**

**Table S1.** Summary of biochemical Mps1 enzyme, BBB values (Schrödinger), and anti-proliferative assay of G13 to G23 and G4 (related to Table 1).

| **Analogue (R_1_)** | **Compound ID** | **Mps1 enzyme assay** | | ***^c^*QPlogBB** | **GBM cytotoxicity IC_50_ (µM)** | | | |
| --- | --- | --- | --- | --- | --- | --- | --- | --- |
|  |  | ***^a^*% Mps1 Inhibition**  **(at 10 µM)** | ***^b^*% Mps1 Inhibition**  **(at 1 µM)** |  | ***^d^*LN229** | ***^e^*LN18** | ***^e^*U87MG** | ***^d^*LN229-TMZ-R** |
| - | G4 | 58.6 ± 22.0 | <1.0 | 0.5 | >10.0 | >10.0 | >10.0 | - |
|  | G13 | 74.4 ± 0.2 | <1.0 | 0.4 | 4.9 ± 0.15 | 5.3 ± 0.89 | >10.0 | - |
|  | G14 | 90.6 ± 4.0 | 26.1 ± 3.7 | -1.0 | 3.7 ± 1.15 | 3.8 ± 0.28 | 7.0 ± 1.02 | - |
|  | G15 | 80.8 ± 17.5 | 34.2 ± 4.5 | -1.1 | >10.0 | >10.0 | >10.0 | - |
|  | G16 | 97.2 ± 6.5 | 30.7 ± 3.0 | -0.1 | 4.8 ± 1.22 | 2.7 ± 0.55 | 4.6 ± 1.62 | - |
|  | G16a | 98.6 ± 9.2 | 40.6 ± 2.0 | -0.5 | >10.0 | >10.0 | >10.0 | - |
|  | G17 | >99.0 | >99.0 | -0.6 | 0.6 ± 0.10 | 0.8 ±0.09 | 1.1 ± 0.78 | 2.0 ± 0.18 |
|  | G18 | >99.0 | 63.9 ± 2.6 | 0.1 | 4.4 ± 0.26 | 7.7 ± 1.45 | 5.6 ± 0.38 | - |
|  | G19 | >99.0 | 39.2 ± 2.8 | 0.3 | 2.5 ± 1.44 | 3.0 ± 0.54 | 6.3 ± 0.08 | - |
|  | G20 | 90.0 ± 8.3 | 42.8 ± 3.4 | 0.1 | 6.2 ± 0.98 | 6.5 ± 0.49 | 6.0 ± 1.23 | - |
|  | G21 | 86.2 ± 3.8 | 67.9 ± 2.3 | -0.7 | 5.4 ± 0.76 | 4.1 ± 0.66 | 4.1 ± 1.10 | - |
|  | G22 | 47.0 ± 6.2 | 42.6 ± 2.3 | 0.1 | 4.9 ± 1.42 | 5.1 ± 0.62 | nd | - |
|  | G23 | 72.7 ± 5.7 | 70.4 ± 2.4 | -0.2 | 2.6 ± 0.21 | 4.5 ± 0.89 | 4.8 ± 0.78 | - |
| - | *^f^*J54 | >99.0 | 38.7 ± 2.8 | 0.8 | 48.7 | 37.8 | 52.3 | nd |
| - | Reversine | >99.0 | 92.1 ± 1.2 | - | - | - | - | - |
| - | MPI-0479605 | >99.0 | 98.7 ± 1.3 | - | - | - | - | - |
| - | Stp | >99.0 | 83.2 ± 0.3 | - | - | - | - | - |
| - | *^f^*TMZ | - | - | nd | 105.9 | nd | 93.6 | nd |

*^a,b^*% Mps1 inhibition determined at 10 µM and 1 µM, respectively, taking DMSO as a vehicle control and Reversine, MPI-0479605, and Staurosporine as a positive control. The enzyme inhibition values represent mean ± SD of two independent experiments (n=2). *^c^*QikProp analysis (Schrödinger software) was utilized to predict compound’s likelihood to cross BBB. Acceptable range for the QPlogBB lies between -1.5 to 3.0. *^d,e^*Cytotoxicity values representing mean ± SD of three and two independent experiments, respectively (n = 3 for *d*, and n = 2 for *e*). *^f^*IC_50_ value of controls (J54 and TMZ) is reported from Priya, B., et al.^[1]^ Stp - staurosporine, TMZ – temozolomide, nd – not determined.

**Table S2.** Summary of Kinome profiling of G17 performed at 1 µM (related to Figure 2D and 2E).

| Kinase | % Inhibition |
| --- | --- |
| ABL | -11.4 |
| ALK | 36.4 |
| ALK[C1156Y] | 29.3 |
| AXL | 10.8 |
| BLK | 3.2 |
| BMX | 2.2 |
| BRK | 10.5 |
| BTK | 2.2 |
| EGFR[T790M] | 3.0 |
| EGFR[T790M/C797S/L858R] | 1.4 |
| EPHA8 | 2.8 |
| FAK | 26.8 |
| FER | 41.8 |
| FES | 21.5 |
| FGFR1 | 10.1 |
| FGFR3 | 26.5 |
| FGR | 3.1 |
| FLT3 | 77.9 |
| FMS | 14.9 |
| FYN[isoform a] | 10.5 |
| IGF1R | 12.1 |
| INSR | 19.7 |
| IRR | 15.0 |
| JAK2 | 52.4 |
| KIT | 11.2 |
| LTK | 50.6 |
| LYNb | 4.1 |
| MET[M1250T] | 3.5 |
| PYK2 | 66.1 |
| RET | 31.3 |
| RET[Y791F] | 23.2 |
| ROS | 7.6 |
| SRC | 8.7 |
| TIE2 | 1.7 |
| YES | 24.6 |
| AKT2 | -0.6 |
| AKT3 | 0.7 |
| AMPKα1/β1/γ1 | 0.1 |
| AurA | 72.8 |
| AurB/INCENP | 19.8 |
| BRAF | 6.8 |
| CaMK1α | 8.2 |
| CaMK1δ | 9.3 |
| CaMK2α | -0.5 |
| CaMK2β | 1.4 |
| CaMK2δ | -0.6 |
| CaMK4 | -0.2 |
| CDK4/CycD1 | 1.2 |
| CDK4/CycD3 | 3.6 |
| CDK7/CycH/MAT1 | 17.5 |
| CGK2 | 3.4 |
| CHK2 | 44.5 |
| CK1ε | 24.7 |
| CK2α1/β | 0.0 |
| CK2α2/β | 1.9 |
| CLK1 | 60.1 |
| CLK2 | 28.1 |
| CLK3 | 2.9 |
| COT | 5.2 |
| DAPK1 | 2.7 |
| DYRK1A | 3.2 |
| DYRK1B | -3.1 |
| DYRK3 | 2.6 |
| Erk1 | 6.8 |
| Erk2 | 11.0 |
| GSK3α | -8.2 |
| GSK3β | -7.9 |
| Haspin | -1.7 |
| HGK | 6.7 |
| IKKε | 8.8 |
| JNK1 | 10.7 |
| JNK2 | 31.7 |
| JNK3 | 30.5 |
| MAP2K1 | 4.8 |
| MAP2K2 | 5.4 |
| MAP2K4 | -7.3 |
| MAP2K5 | 16.0 |
| MAP2K6 | 7.7 |
| MAP2K7 | 9.6 |
| MAP4K2 | 69.6 |
| MAP4K5 | 29.5 |
| MAPKAPK2 | 1.0 |
| MAPKAPK3 | 2.9 |
| MELK | 24.9 |
| MINK | 9.7 |
| MSK1 | 4.5 |
| MSK2 | 2.0 |
| MSSK1 | 1.1 |
| NuaK1 | 85.0 |
| p38α | 4.0 |
| p38γ | 1.2 |
| PAK2 | 1.4 |
| PHKG1 | 20.3 |
| PHKG2 | 0.8 |
| PIM2 | -4.2 |
| PIM3 | -1.4 |
| PKCα | 23.0 |
| PKCβ2 | 17.9 |
| PKD1 | 5.7 |
| PKD2 | 1.9 |
| PKD3 | 1.7 |
| RAF1 | 16.7 |
| RSK2 | 64.6 |
| RSK3 | 78.1 |
| RSK4 | 53.5 |
| SGK | 4.7 |
| skMLCK | 1.4 |
| SRPK1 | 4.1 |
| TSSK1 | 89.8 |
| TSSK2 | 85.9 |

**Figure S1.** Representative Mps1 kinase inhibitors reported in the literature and their biochemical potencies.**^2-7^** While BAY 1217389 and CFI-402257 have demonstrated promising preclinical activity and advanced to clinical evaluation in solid tumor and breast cancer, their therapeutic potential in glioblastoma remains largely unexplored, highlighting the need for the development and evaluation of new Mps1-targeted agents such as G17.

**Figure S2.** (related to Figure 1B and 1C) A) Binding pose overlay of G17 (yellow) and J54 (pink) occupying different regions of Mps1 binding pocket. B) 2D interactions of J54 modelled in the Mps1 kinase domain (PDB ID 6B4W). C) Predicted binding mode of designed G17 in Mps1 cocrystal structure (PDB ID 6B4W) in C) 3D and D) 2D. The Mps1 binding site residues are shown within 5Å. G17 interacts with K529, N606, M671, A651 while maintaining crucial interactions with E603 and G605, the catalytic domain residues.

**Figure S3.** (related to Figure 4A-D) Representative immunocytochemistry images showing γ-H2AX fluorescence intensity in (A) LN229 and (B) LN229-TMZ-R cell lines treated with G17, TMZ (50 µM for LN229 cells and 200 µM for LN229-TMZ-R cells), and 0.1% DMSO for 48 h. (C) and (D) are quantification of γ-H2AX fluorescence intensities of (A) and (B) derived from 20 individual cells of LN229 and LN229-TMZ-R, respectively.

**Figure S4.** (related to Figure 5A-D) Representative Images of A) LN229 and C) LN229-TMZ-R cell colonies formed in presence or absence of G17 and TMZ. B) and D) are quantification of cell colonies from A) and C), respectively, with statistical significance determined by ordinary *one-way ANOVA* relative to the vehicle control.

**Figure S5.** (related to Figure 5H-I) Cell death analysis via dual Hoechst/PI-staining assay in (A) LN229 and (B) LN229-TMZ-R treated with different concentrations of G17, TMZ (50 µM), and 1% DMSO control for 72h. Quantification of PI-positive cells in (C) LN229 and (D) LN229-TMZ-R with statistical significance determined using student’s t-test.

**Chemistry experiments: Methods**

All the chemicals, reagents, catalyst and solvents were purchased from Sigma-Aldrich (Darmstadt, Germany), Merck (Darmstadt, Germany), TCI (Chennai, India) or Finar (Gujarat, India), and used without further purification unless otherwise specified. All the reactions were performed in flame dried double-neck round bottom flasks, sealed tube or schlenk flask with proper rubber septum. The reaction progress was monitored via Merck Thin-Layer Chromatography (TLC silica gel 60 F254) and spots were visualized under UV light (254 nm). Products were purified using Merck silica gel 100-200 mesh size column chromatography using standard protocols.

**Chemistry Instrumentation**

The nuclear magnetic resonance (NMR) spectra of purified compounds were recorded on Bruker Advance III (500 MHz) instrument at 25 °C and referenced against following solvent peaks - CDCl_3_: δ 7.26 ppm and 77.16 ppm for ^1^H and ^13^C, respectively. Chemical shifts are denoted in parts per million (ppm, δ) and referenced downfield from tetramethyl silane. Chemical shifts were referenced as follows: chemical shift, multiplicity (s = singlet, d = doublet, t = triplet, q = quartet, m = multiplet), coupling constant (Hz), and integration. The High Resolution Mass Spectrometry (HRMS) data for all compounds were acquired on Waters Synapt-G2-S ESI-Q-TOF model instrument in positive ion mode with reverse phase C-18 column (particle size 1.7 µm, 2.1 mm x 50 mm) and photodiode array (PDA) detector with method as follows: 95:5 Acetonitrile:water (0.1% formic acid) initial→100% acetonitrile (0.1% formic acid) over 12 minutes, with flow rate of 0.4 mL/min.

The purity of final compounds were estimated via high performance liquid chromatography (HPLC) using Shimadzu Nexera Analytical cum Preparative System (Shimadzu Corporation, Japan) with PDA detector and Shimadzu GIST C18 250 mm column. The compound detections were performed at 210 nm with acetonitrile/water 50:50 isocratic system.

**Chemical synthesis**

A convergent synthetic route was employed for the designed analogues starting with *N*-alkylation of 1 with ethyl morpholine linker affording 3, which upon Pd-catalyzed Buchwald coupling followed by alkaline hydrolysis yielded a key intermediate, 4. Additionally, G4 bearing an acrylamide linker was also synthesized as a negative control (without pyrrolo[2,3-d]pyrimidine ring) using acryloyl chloride under mild conditions. Alternatively, intermediate 5 underwent Suzuki-Miyaura cross coupling with corresponding boronic partners or nucleophilic displacement reaction with cyclic aliphatic amines delivering intermediates G13In to G23In. With these intermediates, final Pd-catalyzed Buchwald-Hartwig coupling was accomplished with 4 and G13In to G23In, followed by SEM deprotection to afford final compounds G13 to G23.

**Scheme S1. Synthesis of phenothiazine-based Mps1 inhibitors**.

***Reagents and conditions.*** a) NaH, dry DMF, 0 °C to r.t., 24 h, and 78%; b) acetamide, Pd_2_dba_3_, X-phos, Cs_2_CO_3_, dry toluene, 110 °C, 6 h, and 67%; c) Ethanolic KOH, 80 °C, overnight, and 84%; d) acryloyl chloride, DIEA, dry DCM, 0 °C to r.t., 2 h, and 66%; e) R_1_-B(OH)_2_, PdCl_2_dppf.DCM, K_2_CO_3_, 1,4-dioxane:H_2_O (9:1), 70 °C, 1 h, and 52-68%; f) R_1_-NH_2_, dry DMSO, 60 °C, 2 h, and 72-88%; g) 4, Pd(OAc)_2_, *rac*-BINAP, Cs_2_CO_3_, dry toluene, 110 °C, 1 h; h) TFA:DCM (1:1), r.t., 1 h, then NH_4_OH in EtOH, 60 °C, 1 h, 35-58% (over 2 steps).

**Detailed experimental procedures**

***Synthesis of 4-(2-(2-chloro-10H-phenothiazin-10-yl)ethyl)morpholine 3:***

To the suspension of NaH (82 mg, 3.43 mmol, 4.0 equiv.) in an oven-dried 250 mL round-bottom flask equipped with a magnetic stirrer bar was added dry DMF (3 mL) dropwise under inert conditions at 0 °C for 10 minutes. Then 2-chloro-10*H*-phenothiazine (200 mg, 0.85 mmol, 1.0 equiv.) dissolved in minimum amount of DMF was added at 0 °C in a drop-wise manner, and the resulting mixture was stirred for 15 mins. Following this, 4-(2-Chloroethyl)morpholine hydrochloride (240 mg, 1.28 mmol, 1.5 equiv.) was then added at 0 °C for 15 mins, and the reaction mixture was left for stirring at room temperature for 24 hours. The reaction progress was monitored on TLC at a 50% EtOAc-Hexane system. After completion, the mixture was dumped into ice-cold water, and ethyl acetate was added to obtain a biphasic layer. Further, the compound was extracted in ethyl acetate (3x), dried with anhydrous Na_2_SO_4_, and evaporated under reduced pressure. The product was purified via silica gel column chromatography (10-50% EtOAc:Hexane) to obtain purified 4-(2-(2-chloro-10*H*-phenothiazin-10-yl)ethyl)morpholine.

Yield: 78% (orange gummy liquid)

^1^H NMR (500 MHz, CDCl_3_) δ 7.16 (td, *J* = 10.0, 5.0 Hz, 1H), 7.12 (dd, *J* = 10.0, 1.5 Hz, 1H), 7.02 (d, *J* = 8.1 Hz, 1H), 6.99 (d, *J* = 2.0 Hz, 1H), 6.94 (td, *J* = 7.6, 1.0 Hz, 1H), 6.90–6.88 (m, 2H), 3.98 (t, *J* = 6.6 Hz, 2H), 3.73 (t, *J* = 4.6 Hz, 4H), 2.76 (t, *J* = 6.7 Hz, 2H), 2.55–2.54 (m, 4H).

^13^C NMR (126 MHz, CDCl_3_) δ 146.4, 144.4, 133.4, 128.0, 127.7, 127.6, 124.7, 123.4, 123.2, 122.5, 116.1, 115.7, 67.1, 56.1, 54.0, 46.2.

HRMS m/z calc. for C_18_H_20_ClN_2_OS^+^ [M + H]^+^ 347.0985 found 347.0991

***Synthesis of N-(10-(2-morpholinoethyl)-10H-phenothiazin-2-yl)acetamide 3’:***

In an oven-dried 50 mL schlenk tube was added 3 (500 mg, 1.44 mmol, 1.0 equiv.) dissolved in dry toluene (7.5 mL). The schlenk tube was fully degassed with argon (30 mins.) and acetamide (174 mg, 2.89 mmol, 2.0 equiv.), X-Phos (103 mg, 0.216 mmol, 0.15 equiv.), cesium carbonate (939 mg, 2.89 mmol, 2.0 equiv.), and Pd_2_(dba)_3_ (99 mg, 0.10 mmol, 0.075 equiv.) were added successively. After addition, the resulting mixture was further flushed with argon for 20 mins, sealed the tube and heated at 110 °C for 6 hours. The reaction progress was monitored via TLC. After completion, the reaction mixture was cooled to room temperature, diluted with ethyl acetate and passed through celite. Following this, the diluted mixture was evaporated in rota vac and subjected to silica gel column chromatography (0-10% Dichloromethane:Methanol) for further purification to obtain the product *N*-(10-(2-morpholinoethyl)-10*H*-phenothiazin-2-yl)acetamide.

Yield: 67% (light-yellow liquid)

^1^H NMR (500 MHz, CDCl_3_) δ 8.65 (s, 1H), 7.45 (s, 1H), 7.09–7.05 (m, 2H), 6.93 (d, *J* = 8.2 Hz, 1H), 6.87–6.81 (m, 3H), 3.85 (t, *J* = 6.5 Hz, 2H), 3.61 (t, *J* = 4.3 Hz, 4H), 2.66 (t, *J* = 6.5 Hz, 2H), 2.41 (bs, 4H), 2.10 (s, 3H).

^13^C NMR (126 MHz, CDCl_3_) δ 168.9, 145.5, 144.5, 137.9, 127.2, 127.1, 124.6, 122.5, 119.5, 115.5, 113.8, 107.9, 66.8, 55.5, 53.6, 45.5, 24.3.

HRMS m/z calc. for C_20_H_24_N_3_O_2_S^+^ [M + H]^+^ 370.1589 found 370.1600

***Synthesis of 10-(2-morpholinoethyl)-10H-phenothiazin-2-amine 4:***

In an oven-dried two-neck 250 mL round-bottom flask equipped with a magnetic stirrer bar and a condenser, 3’ (400 mg, 1.08 mmol, 1.0 equiv.) was added. After that, KOH (1214 mg, 21.68 mmol, 20.0 equiv.) dissolved in ethanol (10 mL) was added slowly, and the resulting mixture was heated at 80 ^°^C overnight. Next day, the resulting mixture was cooled and dumped into ice-cold water and ethyl acetate was added to obtain a biphasic layer. Further, the compound was extracted in ethyl acetate (3x), dried with anhydrous Na_2_SO_4_, and evaporated under reduced pressure. Compound 10-(2-morpholinoethyl)-10H-phenothiazin-2-amine was used without purification.

Yield: 84% (dark brown liquid)

^1^H NMR (500 MHz, CDCl_3_) δ 7.17–7.13 (m, 2H), 6.94–6.88 (m, 3H), 6.33–6.30 (m, 2H), 4.09 (t, *J* = 6.3 Hz, 2H), 3.79 (t, *J* = 4.6 Hz, 4H), 2.95 (bs, 2H), 2.73 (bs, 4H).

^13^C NMR (126 MHz, CDCl_3_) δ 146.7, 146.2, 144.9, 128.4, 127.7, 127.3, 126.6, 122.9, 115.6, 113.4, 110.0, 103.4, 66.3, 55.8, 53.8, 44.8.

HRMS m/z calc. for C_18_H_22_N_3_OS^+^ [M + H]^+^ 328.1484 found 328.1479

***Synthesis of N-(10-(2-morpholinoethyl)-10H-phenothiazin-2-yl)acrylamide G4:***

# To a stirring solution of 4 (200 mg, 0.61 mmol, 1.0 equiv.) dissolved in dry DCM (5 mL) was added DIEA (118.5 mg or 160 µL, 0.91 mmol, 1.5 equiv.) at 0 °C for 15 mins. Next, acryloyl chloride (83 mg or 75 µL, 0.91 mmol, 1.5 equiv.) was added dropwise, and the resulting mixture was stirred at room temperature for 2 hrs. Upon completion, the excess solvent was removed under reduced pressure, and the solid residue was diluted with ethyl acetate and water. The organic components were extracted in ethyl acetate (3x) and reduced under vacuo. The crude obtained was subjected to silica gel column chromatography (0-10% Dichloromethane:Methanol) for further purification to obtain *N*-(10-(2-morpholinoethyl)-10*H*-phenothiazin-2-yl)acrylamide.

# Yield: 66% (light-yellow liquid)

# ^1^H NMR (500 MHz, CDCl_3_) δ 7.95 (s, 1H), 7.45 (s, 1H), 7.16–7.12 (m, 3H), 7.04 (d, *J* = 8.2 Hz, 1H), 6.95–6.88 (m, 2H), 6.43 (d, *J* = 16.9 Hz, 1H), 6.30–6.25 (m, 1H), 5.76 (d, *J* = 10.2 Hz, 1H), 4.08 (t, *J* = 6.8 Hz, 2H), 3.76 (t, *J* = 4.5 Hz, 4H), 2.91 (t, *J* = 6.8 Hz, 2H), 2.69 (bs, 4H).

# ^13^C NMR (126 MHz, CDCl_3_) δ 163.8, 145.3, 144.8, 138.0, 131.3, 128.0, 127.8, 127.7, 125.2, 123.0, 120.3, 115.5, 114.2, 107.8, 66.2, 55.2, 53.5, 44.6.

# HRMS m/z calc. for C_21_H_24_N_3_O_2_S^+^ [M+H]^+^ 382.1589 found 382.1588

***General synthetic procedure for G13In – G16aIn:***

In an oven-dried 25 mL sealed-tube was added R_1_-boronic acids (1.0 equiv.), 3 (1.5 equiv.), K_2_CO_3_ (2.0 equiv.) and 1,4-dioxane:H_2_O (9:1). The tube was flushed with argon for 20 mins and PdCl_2_dppf.DCM (5.0 mol%) was added to it. The resulting mixture was heated at 70 °C for 90 minutes. After completion, the reaction mixture was diluted with ethyl acetate and passed through the celite bed. The diluted mixture was evaporated in rotary evaporator and subjected to silica gel column chromatography for further purification.

***General synthetic procedure for 2-chloro-4-(3-(trifluoromethyl)phenyl)-7-((2-(trimethylsilyl)ethoxy)methyl)-7H-pyrrolo[2,3-d]pyrimidine G13In:***

Yield: 63% (pale yellow liquid) isolated in 10-50% EtOAc:Hexane

^1^H NMR (500 MHz, CDCl_3_): δ 8.41 (s, 1H), 8.34 (d, *J* = 10 Hz, 1H), 7.82 (d, *J* = 10 Hz, 1H), 7.71 (t, *J* = 10 Hz, 1H), 7.49 (d, *J* = 5 Hz, 1H), 6.88 (d, *J* =5 Hz, 1H), 5.70 (s, 2H), 3.61 (t, *J* = 10 Hz, 2H), 0.98 (t, *J* = 5 Hz, 2H), 0.00 (s, 9H).

^13^C{^1^H} NMR (126 MHz, CDCl_3_): δ 157.0, 154.2, 154.1, 137.8, 132.3, 131.5 ( q, *J* = 32.8 Hz), 130.1, 129.6, 127.3 (q, *J* = 2.5 Hz), 126.5, 126.0 (q, *J* = 3.8 Hz), 124.0 (q, *J* = 273.4 Hz), 114.7, 101.6, 73.1, 67.0, 17.8, 1.3.

ESI-MS (Q-TOF) *m/z* [M + H]^+^ calcd for C_19_H_22_ClF_3_N_3_OSi^+^ 428.9352; found 428.1172

***General synthetic procedure for 3-(2-chloro-7-((2-(trimethylsilyl)ethoxy)methyl)-7H-pyrrolo[2,3-d]pyrimidin-4-yl)benzamide G14In:***

Yield: 55% (pale brown liquid) isolated in 10-50% EtOAc:Hexane

^1^H NMR (500 MHz, CDCl_3_): δ 8.55 (s, 1H), 8.26 (d, *J* = 10 Hz, 1H), 8.00 (d, *J* = 10 Hz, 1H), 7.63 (t, *J* = 10 Hz, 1H), 7.43 (d, *J* = 5 Hz, 1H), 6.89 (d, *J* = 5 Hz, 1H), 6.47 (bs, 1H), 6.02 (bs, 1H), 5.65 (s, 2H), 3.57 (t, *J* = 5 Hz, 2H), 0.94 (t, *J* = 5 Hz, 2H), 0.00 (s, 9H).

^13^C{^1^H} NMR (126 MHz, CDCl_3_): δ 168.9, 158.4, 154.1, 154.0, 137.4, 134.3, 132.4, 129.9, 129.8, 129.4, 127.9, 114.7, 102.0, 73.1, 67.0, 17.8, 1.3.

ESI-MS (Q-TOF) *m/z* [M + H]^+^ calcd for C_19_H_24_ClN_4_O_2_Si^+^ 403.962; found 403.1362

***General synthetic procedure for 3-(2-chloro-7-((2-(trimethylsilyl)ethoxy)methyl)-7H-pyrrolo[2,3-d]pyrimidin-4-yl)benzoic acid G15In:***

Yield: 52% (pale brown liquid) isolated in 10-50% EtOAc:Hexane

^1^H NMR (500 MHz, CDCl_3_): δ 8.84 (s, 1H), 8.40 (d, *J* = 10 Hz, 1H), 8.27 (d, *J* = 5 Hz, 1H), 7.67 (t, *J* = 10 Hz, 1H), 7.46 (d, *J* = 5 Hz, 1H), 6.92 (d, *J* = 5 Hz, 1H), 5.67 (s, 2H), 3.59 (t, *J* = 5 Hz, 2H), 0.95 (t, *J* = 10 Hz, 2H), 0.03 (s, 9H).

^13^C{^1^H} NMR (126 MHz, CDCl_3_): δ 171.5, 158.2, 154.2, 154.1, 137.5, 134.3, 132.3, 130.8, 130.1, 129.9, 129.4, 114.7, 101.9, 73.1, 67.0, 17.8, 1.3.

ESI-MS (Q-TOF) *m/z* [M + H]^+^ calcd for C_19_H_23_ClN_3_O_3_Si^+^ 404.9460; found 404.1207

***General synthetic procedure for methyl 3-(2-chloro-7-((2-(trimethylsilyl)ethoxy)methyl)-7H-pyrrolo[2,3-d]pyrimidin-4-yl)benzoate G16In:***

Yield: 59% (pale yellow liquid) isolated in 10-50% EtOAc:Hexane

^1^H NMR (500 MHz, CDCl_3_): δ 8.79 (s, 1H), 8.36 (d, *J* = 10 Hz, 1H), 8.23 (d, *J* = 5 Hz, 1H), 7.66 (t, *J* = 10 Hz, 1H), 7.47 (d, *J* = 5 Hz, 1H), 6.92 (d, *J* = 5 Hz, 1H), 5.69 (s, 2H), 4.01 (s, 3H), 3.61 (t, *J* = 5 Hz, 2H), 0.98 (t, *J* = 5 Hz, 2H), 0.00 (s, 9H).

^13^C{^1^H} NMR (126 MHz, CDCl_3_): δ 166.7, 158.4, 154.1, 154.0, 137.3, 133.4, 131.7, 130.9, 130.2, 129.8, 129.2, 114.7, 101.9, 73.1, 66.9, 52.2, 17.8, 1.4.

ESI-MS (Q-TOF) *m/z* [M + H]^+^ calcd for C_20_H_25_ClN_3_O_3_Si^+^ 418.973; found 418.1354

***General synthetic procedure for 3-(2-chloro-7-((2-(trimethylsilyl)ethoxy)methyl)-7H-pyrrolo[2,3-d]pyrimidin-4-yl)benzonitrile G16aIn:***

Yield: 68% (dark yellow liquid) isolated in 10-50% EtOAc:Hexane

^1^H NMR (500 MHz, CDCl_3_): δ 8.43 (s, 1H), 8.39 (d, *J* = 5 Hz, 1H), 7.84 (d, *J* = 5 Hz, 1H), 7.71 (t, *J* = 5 Hz, 1H), 7.52 (d, *J* = 4 Hz, 1H), 6.89 (d, *J* = 5 Hz, 1H), 5.70 (s, 2H), 3.61 (t, *J* = 10 Hz, 2H), 0.98 (t, *J* = 5 Hz, 2H), 0.00 (s, 9H).

^13^C{^1^H} NMR (126 MHz, CDCl_3_): δ 156.7, 154.1, 138.1, 133.8, 133.1, 132.5, 130.4, 129.9, 118.3, 114.5, 113.3, 101.3, 73.1, 67.0, 17.7, 1.4.

ESI-MS (Q-TOF) *m/z* [M + H]^+^ calcd for C_19_H_22_ClN_4_OSi^+^ 385.947; found 385.1257

# ***General synthetic procedure for G17n to G23In:***

# General procedure for the synthesis of intermediates G17In to G23In:

# In a single neck oven-dried round bottom flask was added 5 (1.0 equiv.), corresponding R_1_-amines (1.2 equiv.), and Cs_2_CO_3_ (2.0 equiv.) in dry DMSO. The mixture was heated at 60 °C for 90 mins. Upon completion, the reaction mixture was cooled to rt and dumped into ice-cold water. The organic layer was separated with ethyl acetate (3x), dried over Na_2_SO_4_, removed under reduced pressure and subjected to silica gel column chromatography for further purification.

# ***(1r,4r)-4-((2-chloro-7-((2-(trimethylsilyl)ethoxy)methyl)-7H-pyrrolo[2,3-d]pyrimidin-4 yl)amino)cyclohexan-1-ol G17In:***

Yield: 79% (pale yellowish-green liquid) isolated in 10-50% EtOAc:Hexane

^1^H NMR (500 MHz, CDCl_3_) δ 7.09 (d, *J* = 4.0 Hz, 1H), 6.43 (d, *J* = 4.0 Hz, 1H), 5.55 (s, 2H), 3.76–3.70 (m, 1H), 3.57 (t, *J* = 8.5 Hz, 2H), 2.40–2.35 (m, 1H), 2.23 (d, *J* = 11.5 Hz, 2H), 2.08 (bs, 2H), 1.59–1.51 (m, 2H), 1.43–1.34 (m, 2H), 0.95 (t, *J* = 8.5 Hz, 2H), 0.00 (s, 9H).

^13^C NMR (126 MHz, CDCl_3_) δ 156.7, 154.1, 151.7, 124.3, 101.1, 99.5, 72.8, 69.8, 66.4, 60.5, 33.9, 31.2, 17.7, -1.4.

HRMS m/z calc. for [M + H]^+^ C_18_H_30_ClN_4_O_2_Si^+^ 397.1827 found 397.1823

# ***2-chloro-4-(piperazin-1-yl)-7-((2-(trimethylsilyl)ethoxy)methyl)-7H-pyrrolo[2,3-d]pyrimidine G18In:***

Yield: 72% (brown gummy liquid) isolated in 0-5% Dichloromethane:Methanol

^1^H NMR (500 MHz, CDCl_3_) δ 7.10 (d, *J* = 3.5 Hz, 1H), 6.55 (d, *J* = 3.5 Hz, 1H), 5.57 (s, 2H), 4.03 (t, *J* = 5.0 Hz, 4H), 3.56 (t, *J* = 8.5 Hz, 2H), 3.11 (t, *J* = 5.0 Hz, 4H), 0.95 (t, *J* = 8.0 Hz, 2H), 0.00 (s, 9H).

^13^C NMR (126 MHz, CDCl_3_) δ 157.5, 153.7, 153.3, 123.8, 102.3, 101.5, 73.0, 66.5, 46.3, 45.7, 17.8, -1.3.

HRMS m/z calc. for C_16_H_27_ClN_5_OSi^+^ [M + H]^+^ 368.1673 found 368.1661

# ***2-chloro-4-(4-methylpiperazin-1-yl)-7-((2-(trimethylsilyl)ethoxy)methyl)-7H-pyrrolo[2,3-d]pyrimidine G19In:***

Yield: 77% (dark yellow liquid) isolated in 0-5% Dichloromethane:Methanol

^1^H NMR (500 MHz, CDCl_3_) δ 7.09 (d, *J* = 3.5 Hz, 1H), 6.55 (d, *J* = 3.5 Hz, 1H), 5.57 (s, 2H), 4.03 (t, *J* = 5.0 Hz, 4H), 3.56 (t, *J* = 8.5 Hz, 2H), 2.60 (t, *J* = 5.0 Hz, 4H), 2.40 (s, 3H), 0.95 (t, *J* = 8.5 Hz, 2H), 0.00 (s, 9H).

^13^C NMR (126 MHz, CDCl_3_) δ 157.5, 153.7, 153.3, 123.7, 102.3, 101.5, 73.0, 66.5, 54.9, 46.1, 45.5, 17.8, -1.3.

HRMS m/z calc. for C_17_H_29_ClN_5_OSi^+^ [M + H]^+^ 382.183 found 382.1825

***2-chloro-4-(piperidin-1-yl)-7-((2-(trimethylsilyl)ethoxy)methyl)-7H-pyrrolo[2,3-d]pyrimidine G20In:***

Yield: 78% (pale brown liquid) isolated in 10-50% EtOAc:Hexane

^1^H NMR (500 MHz, CDCl_3_) δ 7.06 (d, *J* = 3.5 Hz, 1H), 6.55 (d, *J* = 3.5 Hz, 1H), 5.56 (s, 2H), 3.93 (t, *J* = 5.5 Hz, 4H), 3.57 (t, *J* = 8.0 Hz, 2H), 1.77–1.73 (m, 6H), 0.95 (t, *J* = 8.5 Hz, 2H), 0.00 (s, 9H).

^13^C NMR (126 MHz, CDCl_3_) δ 157.4, 153.8, 153.2, 123.2, 102.6, 101.3, 72.9, 66.4, 47.1, 26.0, 24.6, 17.8, -1.4.

HRMS m/z calc. for C_17_H_28_ClN_4_OSi^+^ [M + H]^+^ 367.1721 found 367.1729

# ***1-(2-chloro-7-((2-(trimethylsilyl)ethoxy)methyl)-7H-pyrrolo[2,3-d]pyrimidin-4-yl)piperidin-4-ol G21In:***

Yield: 76% (pale yellow liquid) isolated in 10-50% EtOAc:Hexane

^1^H NMR (500 MHz, CDCl_3_) δ 7.05 (d, *J* = 3.0 Hz, 1H), 6.52 (d, *J* = 3.0 Hz, 1H), 5.52 (s, 2H), 4.37 (d, *J* = 13.0 Hz, 2H), 4.04 (bs, 1H), 3.60–3.51 (m, 4H), 3.13–3.09 (m, 1H), 2.02 (bs, 2H), 1.66 (bs, 2H), 0.88 (t, *J* = 6.0 Hz, 2H), -0.04 (s, 9H).

^13^C NMR (126 MHz, CDCl_3_) δ 157.4, 153.8, 153.3, 123.6, 102.5, 101.4, 73.0, 67.5, 66.6, 43.3, 22.8, 17.9, -1.3.

HRMS m/z calc. for C_17_H_28_ClN_4_O_2_Si^+^ [M + H]^+^ 383.167 found 383.1659

# ***4-(2-chloro-7-((2-(trimethylsilyl)ethoxy)methyl)-7H-pyrrolo[2,3-d]pyrimidin-4-yl)morpholine G22In:***

Yield: 80% (pale yellow liquid) isolated in 10-50% EtOAc:Hexane

^1^H NMR (500 MHz, CDCl_3_) δ 7.11 (d, *J* = 3.5 Hz, 1H), 6.54 (d, *J* = 3.5 Hz, 1H), 5.57 (s, 2H), 3.98 (t, *J* = 4.5 Hz, 4H), 3.86 (t, *J* = 4.5 Hz, 4H), 3.57 (t, *J* = 8 Hz, 2H), 0.95 (t, *J* = 8.0 Hz, 2H), 0.00 (s, 9H).

^13^C NMR (126 MHz, CDCl_3_) δ 157.5, 153.5, 153.2, 123.8, 102.0, 101.3, 72.8, 66.6, 66.4, 45.8, 17.7, -1.4.

HRMS m/z calc. for C_16_H_26_ClN_4_O_2_Si^+^ [M + H]^+^ 369.1514 found 369.1523

# ***2-chloro-N-cyclohexyl-7-((2-(trimethylsilyl)ethoxy)methyl)-7H-pyrrolo[2,3-d]pyrimidin-4-amine G23In:***

Yield: 88% (pale green liquid) isolated in 10-50% EtOAc:Hexane

1H NMR (500 MHz, CDCl_3_) δ 7.07 (d, *J* = 3.5 Hz, 1H), 6.43 (d, *J* = 3.5 Hz, 1H), 5.55 (s, 2H), 4.10 (bs, 1H), 3.57 (t, *J* = 8.0 Hz, 2H), 2.15–2.12 (m, 2H), 1.83–1.79 (m, 2H), 1.72–1.69 (m, 1H), 1.52–1.44 (m, 2H), 1.34–1.25 (m, 4H), 0.95 (t, *J* = 8.5 Hz, 2H), 0.00 (s, 9H).

13C NMR (126 MHz, CDCl_3_) δ 156.7, 154.2, 151.9, 124.0, 101.0, 99.6, 72.9, 66.4, 33.5, 29.8, 25.6, 24.8, 17.8, -1.4.

HRMS m/z calc. for C_18_H_30_ClN_4_OSi^+^ [M+H]^+^ 381.1877 found 381.1876

*General synthetic procedure for G13 to G23:*

In an oven-dried 50 mL schlenk tube, corresponding halides from G13In to G23In (1.0 equiv.) dissolved in dry toluene was added. The schlenk tube was fully degassed with argon (30 mins.) and 4 (1.5 equiv.), *rac*-BINAP (10.0 mol%), cesium carbonate (2.0 equiv.), and Pd(OAc)_2_ (10.0 mol%) were added successively in an inert condition. The resulting mixture was further flushed with argon for 20 mins; the tube was sealed and heated at 110 °C for 90 minutes. The reaction progress was monitored via TLC. After completion, the reaction mixture was cooled, diluted with ethyl acetate and filtered through celite. Following this, the diluted mixture was evaporated in rota vac, and the crude was used without purification in the next deprotection step.

The SEM-protected crude derivatives obtained from above procedure was treated with trifluoroacetic acid:DCM (1:1) mixture and stirred for 2 hours at room temperature. After that, excess TFA:DCM mixture was removed under reduced pressure, and the resulting mixture was diluted with ethanol and NH_4_OH (3x volume of ethanol). The mixture was heated at 60 °C in an oil bath for 1 hour, diluted with saturated brine and extracted with ethyl acetate (3x). Excess solvent was removed under reduced pressure, and the product was purified using silica gel column chromatography.

***10-(2-morpholinoethyl)-N-(4-(3-(trifluoromethyl)phenyl)-7H-pyrrolo[2,3-d]pyrimidin-2-yl)-10H-phenothiazin-2-amine G13:***

Yield: 52% (dark yellow liquid) isolated in 0-10% Dichloromethane:Methanol

^1^H NMR (500 MHz, CDCl_3_): δ 9.97 (bs, 1H), 8.38 (s, 1H), 8.28 (d, *J* = 10 Hz, 1H), 7.89 (s, 1H), 7.77 (d, *J* =10 Hz, 1H), 7.67 (t, *J* = 7.5 Hz, 1H), 7.30 (s, 1H), 7.17–7.12 (m, 2H), 7.05 (d, *J* = 10 Hz, 1H), 7.02–7.01 (m, 1H), 6.98 (dd, *J* = 8.0, 2.0 Hz, 1H), 6.93 (td, *J* = 7.5, 1.0 Hz, 1H), 6.88 (d, *J* = 5.0 Hz, 1H), 6.62 ( dd, *J* = 3.5, 1.5 Hz, 1H), 4.14 (t, *J* = 6.5 Hz, 2H), 3.71 (t, *J* = 4.5 Hz, 4H), 2.90 (t, *J* = 7 Hz, 2H), 2.60 (bs, 4H).

^13^C{^1^H} NMR (126 MHz, CDCl_3_): δ 156.8, 156.0, 154.4, 145.6, 145.0, 140.1, 139.0, 131.9, 131.2 (q, *J* = 31.5 Hz), 129.2, 127.6, 127.4, 127.2, 126.5 (q, *J* = 3.8 Hz), 125.7, 125.6 (q, *J* = 3.8 Hz), 124.1 (q, *J* = 272.2 Hz), 123.5, 122.7, 116.9, 115.6, 115.3, 113.2, 109.7, 107.1, 101.1, 66.5, 56.0, 53.7, 44.7.

HRMS (ESI-TOF) *m/z* [M + H]^+^ calcd for C_31_H_28_F_3_N_6_OS^+^ 589.1997; found 589.2008

***3-(2-((10-(2-morpholinoethyl)-10H-phenothiazin-2-yl)amino)-7H-pyrrolo[2,3-d]pyrimidin-4-yl)benzamide G14:***

Yield: 38% (dark brown liquid) isolated in 0-10% Dichloromethane:Methanol

^1^H NMR (500 MHz, DMSO-d6): δ 11.72 (s, 1H), 9.45 (s, 1H), 8.63 (s, 1H), 8.28 (d, *J* = 8.0 Hz, 1H), 8.16 (s, 1H), 8.04 (d, *J* = 8.0 Hz, 1H), 7.86 (s, 1H), 7.69 (t, *J* = 7.5 Hz, 1H), 7.53–7.51 (m, 2H), 7.34 (t, *J* = 2.5 Hz, 1H), 7.20 (t, *J* = 8.0 Hz, 1H), 7.15–7.11 (m, 2H), 7.02 (d, *J* = 10 Hz, 1H), 6.93 (t, *J* = 7.0 Hz, 1H), 6.70 (d, *J* = 1.5 Hz, 1H), 4.01 (bs, 2H), 3.48 (bs, 4H), 2.65 (s, 2H), 2.36 (s, 4H).

^13^C{^1^H} NMR (126 MHz, DMSO-d6): δ 167.7, 156.1, 155.7, 154.1, 145.0, 144.9, 141.6, 138.3 135.0, 132.5, 131.1, 128.74, 128.69, 127.7, 127.4, 127.0, 126.8, 124.8, 122.3, 115.8, 113.4, 112.4, 109.1, 106.1, 100.1, 66.1, 55.4, 53.4, 45.1.

HRMS (ESI-TOF) *m/z* [M + H]^+^ calcd for C_31_H_30_N_7_O_2_S^+^ 564.2182; found 564.2203

***3-(2-((10-(2-morpholinoethyl)-10H-phenothiazin-2-yl)amino)-7H-pyrrolo[2,3-d]pyrimidin-4-yl)benzoic acid G15:***

Yield: 35% (dark brown gummy liquid) isolated in 0-10% Dichloromethane:Methanol

^1^H NMR (500 MHz, DMSO-d6): δ 11.75 (s, 1H), 9.47 (s, 1H), 8.75 (s, 1H), 8.38 (d, *J* = 8.0 Hz, 1H), 8.12 (d, *J* = 7.5 Hz, 1H), 7.84 (s, 1H), 7.74 (t, *J* = 7.5 Hz, 1H), 7.53 (d, *J* = 8.0 Hz 1H), 7.35 (t, *J* = 2.0 Hz, 1H), 7.21–7.18 (m, 1H), 7.15–7.10 (m, 2H), 7.02 (d, *J* = 10 Hz, 1H), 6.93 (t, *J* = 7.0 Hz, 1H), 6.68 (dd, *J* = 3.5, 1.5 Hz, 1H), 4.01 (t, *J* = 6.5 Hz, 2H), 3.47 (t, *J* = 4.5 Hz, 4H), 2.66 (t, *J* = 6.5 Hz 2H), 2.37 (bs, 4H).

^13^C{^1^H} NMR (126 MHz, DMSO-d6): δ 167.2, 155.7, 155.5, 154.2, 145.0, 144.9, 141.6, 138.4, 132.6, 130.6, 130.1, 129.2, 129.1, 127.4, 127.0, 126.8, 125.0, 124.3, 122.3, 115.8, 113.5, 112.4, 109.0, 106.0, 99.9, 66.1, 55.3, 53.4, 45.0.

HRMS (ESI-TOF) *m/z* [M + H]^+^ calcd for C_31_H_29_N_6_O_3_S^+^ 565.2022; found 565.2024

***methyl 3-(2-((10-(2-morpholinoethyl)-10H-phenothiazin-2-yl)amino)-7H-pyrrolo[2,3-d]pyrimidin-4-yl)benzoate G16:***

Yield: 56% (pale yellow liquid) isolated in 0-10% Dichloromethane:Methanol

^1^H NMR (500 MHz, CDCl_3_): δ 10.10 (s, 1H), 8.78 (s, 1H), 8.29 (td, *J* = 8.0, 1.0 Hz, 1H), 8.18 (td, *J* = 8.0, 1.0 Hz, 1H), 7.70 (s, 1H), 7.62 (t, *J* = 8.0 Hz, 1H), 7.42 (s, 1H), 7.14 (dd, *J* = 7.5, 1.5 Hz, 1H), 7.08 (dt, *J* = 8.5, 1.5 Hz, 1H), 7.02 (s, 2H), 6.89 (dt, *J* = 7.5, 1.0 Hz, 1H), 6.85–6.84 (m, 1H), 6.82 (d, *J* = 8.0 Hz, 1H), 6.61 (dd, *J* = 3.5, 1.5 Hz, 1H), 4.04 (t, *J* = 7.0 Hz, 2H), 3.98 (s, 3H), 3.65 (t, *J* = 4.0 Hz, 4H), 2.78 (t, *J* = 7.0 Hz, 2H), 2.49 (bs, 4H).

^13^C{^1^H} NMR (126 MHz, CDCl_3_): δ 156.13, 156.08, 155.9, 155.8, 154.7, 153.2, 145.8, 145.6, 145.13, 145.05, 140.3, 140.0, 139.6, 139.4, 133.3, 133.2, 132.94, 132.91, 132.40, 132.36, 129.68, 129.65, 127.8, 127.6, 127.49, 127.45, 127.32, 127.29, 125.69, 125.65, 123.8, 123.7 122.9, 122.8, 118.8, 118.7, 117.2, 116.7, 115.5, 115.2, 113.3, 113.2, 113.1, 112.6, 110.1, 109.7, 107.2, 106.4, 101.1, 101.0, 66.9, 66.0, 56.1, 56.0, 54.3, 54.0, 45.2, 44.8.

HRMS (ESI-TOF) *m/z* [M + H]^+^ calcd for C_32_H_31_N_6_O_3_S^+^ 579.2178; found 579.2172

***3-(2-((10-(2-morpholinoethyl)-10H-phenothiazin-2-yl)amino)-7H-pyrrolo[2,3-d]pyrimidin-4-yl)benzonitrile G16a:***

Yield: 58% (pale yellow liquid) isolated in 0-10% Dichloromethane:Methanol

G16a was found as a pair of rotamer.

^1^H NMR (500 MHz, CDCl_3_): δ 10.04–8.50 (m, 1H), 8.36–8.27 (m, 2H), 7.79–7.33(m, 3H), 7.21–7.03 (m, 3H), 7.02–6.88 (m, 4H), 6.66–5.77 (m, 2H), 4.21–4.10 (m, 2H), 3.71–3.68 (m, 4H), 2.93–2.83 (m, 2H), 2.55 (s, 4H).

^13^C{^1^H} NMR (126 MHz, CDCl_3_): δ 166.9, 157.5, 156.1, 154.5, 145.8, 145.1, 140.1, 138.7, 133.1, 131.1, 130.9, 130.0, 129.0, 127.6, 127.5, 127.3, 125.6, 123.4, 122.7, 117.1, 115.5, 113.5, 110.0, 107.4, 101.4, 66.8, 56.0, 53.9, 52.5, 45.2.

HRMS (ESI-TOF) *m/z* [M + H]^+^ calcd for C_31_H_28_N_7_OS^+^ 546.2076; found 546.2081

***(1r,4r)-4-((2-((10-(2-morpholinoethyl)-10H-phenothiazin-2-yl)amino)-7H-pyrrolo[2,3-d]pyrimidin-4-yl)amino)cyclohexan-1-ol G17:***

Yield: 51% (pale green gummy liquid) isolated in 0-10% Dichloromethane:Methanol

^1^H NMR (500 MHz, CDCl_3_) δ 9.71 (s, 1H), 7.51 (d, *J* = 1.5 Hz, 1H), 7.14–7.08 (m, 3H), 7.00 (d, *J* = 8.0, 2H), 6.90 (td, *J* = 8.5, 1.0 Hz, 1H), 6.83 (d, *J* = 8 Hz, 1H), 6.63 (d, *J* = 3.5 Hz, 1H), 6.16 (s, *J* = 3.5 Hz, 1H), 4.78 (bs, 1H), 4.04, (t, *J* = 7.0 Hz, 3H), 3.69 (t, *J* = 4.5 Hz, 4H), 2.80 (t, *J* = 7.0 Hz, 2H), 2.54 (t, *J* = 4.0 Hz, 4H), 2.20 (d, *J* = 11.0 Hz, 2H), 2.05–2.03 (m, 4H), 1.48 (qd, *J* = 16.0, 3.0 Hz, 2H), 1.33 (td, *J* = 14.5, 3.5 Hz, 2H).

^13^C NMR (126 MHz, CDCl_3_) δ 156.4, 156.2, 152.3,145.7, 145.3, 140.7, 127.6, 127.4, 127.2, 125.9, 122.6, 118.5, 116.6, 115.5, 113.6, 107.5, 98.7, 97.6, 70.1, 66.9, 56.1, 54.0, 49.2, 45.3, 34.3, 31.4

HRMS m/z calc. for C_30_H_36_N_7_O_2_S^+^ [M + H]^+^ 558.2651 found 558.2650

***10-(2-morpholinoethyl)-N-(4-(piperazin-1-yl)-7H-pyrrolo[2,3-d]pyrimidin-2-yl)-10H-phenothiazin-2-amine G18:***

# Yield: 39% (brown gummy liquid) isolated in 0-10% Dichloromethane:Methanol (in 0-10% triethylamine)

# ^1^H NMR (500 MHz, CDCl_3_) δ 10.5 (bs, 1H), 7.42 (s, 1H), 7.11–7.06 (m, 3H), 6.99–6.95 (m, 2H), 6.87 (t, *J* = 7.5 Hz, 1H), 6.77 (d, *J* = 8.0 Hz, 1H), 6.55 (d, *J* = 3.0 Hz, 1H), 6.21 (d, *J* = 3.0 Hz, 1H), 3.93 (bs, 6H), 3.63 (s, 4H), 3.04 (d, *J* = 7.0 Hz, 2H), 2.70 (t, *J* = 6.5 Hz, 2H), 2.64 (bs, 2H), 2.47 (bs, 4H).

# ^13^C NMR (126 MHz, CDCl_3_) δ 157.4, 155.2, 153.6,145.8, 145.0, 140.5, 127.5, 127.4, 127.2, 125.6, 122.5, 118.7, 116.6, 115.4, 113.6, 107.4, 101.6, 97.8, 66.8, 55.9, 53.9, 45.5, 45.2, 32.0

# HRMS m/z calc. for C_28_H_33_N_8_OS^+^ [M + H]^+^ 529.2498 found 529.2515

***N-(4-(4-methylpiperazin-1-yl)-7H-pyrrolo[2,3-d]pyrimidin-2-yl)-10-(2-morpholinoethyl)-10H-phenothiazin-2-amine G19:***

Yield: 44% (dark brown liquid) isolated in 0-15% Dichloromethane:Methanol

^1^H NMR (500 MHz, CDCl_3_) δ 10.37 (bs, 1H), 7.50 (bs, 1H), 7.13–7.07 (m, 3H), 6.97 (bs, 2H), 6.88 (td, *J* = 8.0, 1.0 Hz, 1H), 6.77 (d, *J* = 8.0 Hz, 1H), 6.55 (d, *J* = 3.5 Hz, 1H), 6.28 (d, *J* = 3.5 Hz, 1H), 3.97 (d, *J* = 7.0 Hz, 2H), 3.94 (t, *J* = 5.0 Hz, 4H), 3.65 (t, *J* = 4.5 Hz, 4H), 2.72 (t, *J* = 6.5 Hz, 2H), 2.53 (t, *J* = 5.0 Hz, 4H), 2.48 (bs, 4H), 2.35 (s, 3H).

^13^C NMR (126 MHz, CDCl_3_) δ 157.6, 155.3, 153.6,145.8, 145.1, 140.6, 127.5, 127.4, 127.1, 125.7, 122.5, 118.4, 116.4, 115.4, 113.5, 107.4, 101.9, 97.8, 66.9, 55.9, 55.0, 53.9, 46.2, 45.5, 45.1

HRMS m/z calc. for C_29_H_35_N_8_OS^+^ [M + H]^+^ 543.2655 found 543.2617

***10-(2-morpholinoethyl)-N-(4-(piperidin-1-yl)-7H-pyrrolo[2,3-d]pyrimidin-2-yl)-10H-phenothiazin-2-amine G20:***

Yield: 48% (dark yellow liquid) isolated in 0-10% Dichloromethane:Methanol

^1^H NMR (500 MHz, CDCl_3_) δ 10.47 (bs, 1H), 7.50 (d, *J* = 1.5 Hz, 1H), 7.22 (bs, 1H), 7.12 (dd, *J* = 7.5, 1.5 Hz, 1H), 7.08 (td, *J* = 9.0, 1.5 Hz, 1H), 7.00–6.97 (m, 2H), 6.88 (td, *J* = 8.5, 1.0 Hz, 1H), 6.58 (d, *J* = 8.0 Hz, 1H), 6.51 (d, *J* = 4.0 Hz, 1H), 6.29 (d, *J* = 4.0 Hz, 1H), 3.95 (t, *J* = 3.5 Hz, 2H), 3.87 (t, *J* = 5.5 Hz, 4H), 3.64 (t, *J* = 5.0 Hz, 4H), 2.69 (t, *J* = 7.0 Hz, 2H), 2.46 (bs, 4H), 1.73–1.68 (m, 6H).

^13^C NMR (126 MHz, CDCl_3_) δ 157.5, 155.4, 153.4,145.8, 145.1, 140.8, 127.5, 127.4, 127.1, 125.7, 122.4, 118.0, 116.2, 115.5, 113.5, 107.3, 102.2, 97.7, 66.9, 55.9, 53.9, 47.0, 45.1, 26.1, 24.9

HRMS m/z calc. for C_29_H_34_N_7_OS^+^ [M + H]^+^ 528.2546 found 528.2567

***1-(2-((10-(2-morpholinoethyl)-10H-phenothiazin-2-yl)amino)-7H-pyrrolo[2,3-d]pyrimidin-4 l)piperidin-4-ol G21:***

Yield: 44% (yellow liquid) isolated in 0-10% Dichloromethane:Methanol

^1^H NMR (500 MHz, CDCl_3_) δ 10.58 (bs, 1H), 7.44 (bs, 1H), 7.30 (bs, 1H), 7.10 (dd, *J* = 7.5, 1.5 Hz, 1H), 7.06 (td, *J* = 9.0, 1.5 Hz, 1H), 6.95 (s, 2H), 6.86 (td, *J* = 8.5, 0.5 Hz, 1H), 6.73 (d, *J* = 8.0 Hz, 1H), 6.49 (d, *J* = 3.5 Hz, 1H), 6.25 (d, *J* = 3.5 Hz, 1H), 6.37–6.34 (m, 2H), 3.93–3.88 (m, 3H), 3.62 (t, *J* = 4.5 Hz, 4H), 3.36 (td, *J* = 12.5, 2.0 Hz, 2H), 3.22 (bs, 1H), 2.66 (t, *J* = 7.0 Hz, 2H), 2.43 (bs, 4H), 1.94–1.92 (m, 2H), 1.59–1.52 (m, 2H).

^13^C NMR (126 MHz, CDCl_3_) δ 157.3, 155.3, 153.5,145.8, 145.0, 140.7, 127.5, 127.4, 125.1, 125.7, 122.5, 118.3, 116.3, 115.4, 113.6, 107.4, 101.9, 97.7, 67.7, 66.8, 55.8, 53.8, 45.0, 43.5, 34.4

HRMS m/z calc. for C_29_H_34_N_7_O_2_S^+^ [M + H]^+^ 544.2495 found 544.2501

***N-(4-morpholino-7H-pyrrolo[2,3-d]pyrimidin-2-yl)-10-(2-morpholinoethyl)-10H-phenothiazin-2-amine G22:***

# Yield: 54% (brownish yellow liquid) isolated in 0-10% Dichloromethane:Methanol

# ^1^H NMR (500 MHz, CDCl_3_) δ 10.95 (bs, 1H), 7.35 (d, *J* = 28 Hz, 2H), 7.12 (dd, *J* = 7.5, 1.5 Hz, 1H), 7.06 (td, *J* = 8.5, 1.5 Hz, 1H), 6.98 (bs, 2H), 6.88 (td, *J* = 8.5, 1.0 Hz, 1H), 6.70 (d, *J* = 8.0 Hz, 1H), 6.36 (d, *J* = 2.0 Hz, 1H), 6.21 (d, *J* = 3.0 Hz, 1H), 3.89 (t, *J* = 4.5 Hz, 6H), 3.81 (t, *J* = 5.0 Hz, 4H), 3.61 (t, *J* = 4.5 Hz, 4H), 2.61 (t, *J* = 7.0 Hz, 2H), 2.41 (bs, 4H).

# ^13^C NMR (126 MHz, CDCl_3_) δ 157.7, 155.2, 153.7,145.9, 144.9, 140.3, 127.4, 127.0, 125.5, 122.4, 118.6, 116.7, 115.3, 113.8, 107.7, 101.5, 97.8, 66.84, 66.81, 55.75, 53.8, 45.9, 45.1

# HRMS m/z calc. for C_28_H_32_N_7_O_2_S^+^ [M + H]^+^ 530.2338 found 530.2322

***N4-cyclohexyl-N2-(10-(2-morpholinoethyl)-10H-phenothiazin-2-yl)-7H-pyrrolo[2,3-d]pyrimidine-2,4-diamine G23:***

Yield: 57% (greenish yellow liquid) isolated in 0-10% Dichloromethane:Methanol

^1^H NMR (500 MHz, CDCl_3_) δ 10.74 (bs, 1H), 7.37 (d, *J* = 25 Hz, 2H), 7.15 (d, *J* = 8.0, 1H), 7.12 (dd, *J* = 7.5, 1.5 Hz, 1H), 7.06 (td, *J* = 9.0, 1.5 Hz, 1H), 6.99 (d, *J* = 8.5 Hz, 1H), 6.87 (t, *J* = 7.5 Hz, 1H), 6.71 (d, *J* = 8.0 Hz, 1H), 6.35 (d, *J* = 3.0 Hz, 1H), 6.10 (d, *J* = 3.5 Hz, 1H), 4.93 (bs, 1H), 4.03 (bs, 1H), 3.88 (t, *J* = 7.0 Hz, 2H), 3.61 (t, *J* = 4.0 Hz, 4H), 2.61 (t, *J* = 6.5 Hz, 2H), 2.41 (bs, 4H), 2.10 (d, *J* = 9.5 Hz, 2H), 1.79–1.76 (m, 2H), 1.68 – 1.65 (m, 1H), 1.45 – 1.37 (m, 2H), 1.28 – 1.18 (m, 3H).

^13^C NMR (126 MHz, CDCl_3_) δ 156.3, 156.0, 145.8, 145.0, 140.6, 127.4, 127.1, 125.6, 122.4, 118.6, 116.4, 115.4, 113.7, 107.6, 66.9, 55.8, 53.8, 45.2, 33.7, 25.7, 25.2

HRMS m/z calc. for C_30_H_36_N_7_OS^+^ [M + H]^+^ 542.2702 found 542.2741

**^1^H NMR spectrum, ^13^C NMR spectrum, and HPLC analysis of final compounds.**

**^1^H NMR and ^13^C NMR spectrum of compound G4**

**HPLC analysis of compound G4**

mAU

**Chromatogram**

**min**

**0.0**

**2.5**

**5.0**

**7.5**

**10.0**

**0**

**500**

**1000**

**1500**

**2000**

**2500**

**3000**

**2**

**.**

**389**

**4**

**.**

**328**

PDA Ch1 210nm

Peak#

1

2

Total

Ret. Time

2.389

4.328

Area%

99.855

0.145

100.000

**^1^H NMR and ^13^C NMR spectrum of compound G13**

**HPLC analysis of compound G13**

mAU

**Chromatogram**

**min**

**0.0**

**2.5**

**5.0**

**7.5**

**10.0**

**0**

**250**

**500**

**750**

**1000**

**1250**

**1500**

**1750**

**2000**

**2250**

**2500**

**2**

**.**

**283**

PDA Ch1 210nm

Peak#

1

Total

Ret. Time

2.283

Area%

100.000

100.000

**^1^H NMR and ^13^C NMR spectrum of compound G14**

**HPLC analysis of compound G14**

mAU

**Chromatogram**

**min**

**0.0**

**2.5**

**5.0**

**7.5**

**10.0**

**0**

**250**

**500**

**750**

**1000**

**1250**

**1500**

**1750**

**2000**

**2**

**.**

**330**

**3**

**.**

**311**

PDA Ch1 210nm

Peak#

1

2

Total

Ret. Time

2.330

3.311

Area%

93.717

6.283

100.000

**^1^H NMR and ^13^C NMR spectrum of compound G15**

**HPLC analysis of compound G15**

mAU

**^1^H NMR and ^13^C NMR spectrum of compound G16**

**HPLC analysis of compound G16**

mAU

**^1^H NMR and ^13^C NMR spectrum of compound G16a (a pair of rotamer)**

**HPLC analysis of compound G16a**

mAU

**^1^H NMR and ^13^C NMR spectrum of compound G17**

**HPLC analysis of compound G17**

mAU

**Chromatogram**

**min**

**0.0**

**2.5**

**5.0**

**7.5**

**10.0**

**0**

**500**

**1000**

**1500**

**2000**

**2500**

**3000**

**2**

**.**

**385**

PDA Ch1 210nm

Peak#

1

Total

Ret. Time

2.385

Area%

100.000

100.000

**^1^H NMR and ^13^C NMR spectrum of compound G18**

**HPLC analysis of compound G18**

mAU

**^1^H NMR and ^13^C NMR spectrum of compound G19**

**HPLC analysis of compound G19**

mAU

**^1^H NMR and ^13^C NMR spectrum of compound G20**

**HPLC analysis of compound G20**

mAU

**^1^H NMR and ^13^C NMR spectrum of compound G21**

**HPLC analysis of compound G21**

mAU

**^1^H NMR and ^13^C NMR spectrum of compound G22**

**HPLC analysis of compound G22**

mAU

**^1^H NMR and ^13^C NMR spectrum of compound G23**

**HPLC analysis of compound G23**

mAU

**References**

[1] Priya, B., Chhabria, D., Dhongdi, J.M., and Kirubakaran, S. (2024). A novel approach to investigate the combinatorial effects of TLK1 inhibitors with temozolomide for glioblastoma therapy. *Bioorg. Chem.* 151, 107643.

[2] Atrafi, F., Boix, O., Subbiah, V., Diamond, J. R., Chawla, S. P., Tolcher, A. W., LoRusso, P. M., Eder, J. P., Gutierrez, M., Sankhala, K., et al. (2021). A Phase I Study of an MPS1 Inhibitor (BAY 1217389) in Combination with Paclitaxel Using a Novel Randomized Continual Reassessment Method for Dose Escalation Available to Purchase. *Clin. Cancer Res.* 27, 6366−6375. <https://doi.org/10.1158/1078-0432.CCR-20-4185>

[3] Mason, J. M., Wei, X., Fletcher, G. C., Kiarash, R., Brokx, R., Hodgson, R., Beletskaya, I., Bray, M. R., and Mak, T. W. (2017) Functional characterization of CFI-402257, a potent and selective Mps1/TTK kinase inhibitor, for the treatment of cancer. *Proc. Natl. Acad. Sci. U. S. A.* 114, 3127−3132. <https://doi.org/10.1073/pnas.1700234114>

[4] Liu, Y.; Laufer, R.; Patel, N. K.; Ng, G.; Sampson, P. B.; Li, S. W.; Lang, Y.; Feher, M.; Brokx, R.; Beletskaya, I.; et al. (2016). Discovery of Pyrazolo[1,5-a]pyrimidine TTK Inhibitors: CFI-402257 is a Potent, Selective, Bioavailable Anticancer Agent. *ACS Med. Chem. Lett.* 7, 671−675. <https://doi.org/10.1021/acsmedchemlett.5b00485>

[5] Uitdehaag, J. C. M.; de Man, J.; Willemsen-Seegers, N.; Prinsen, M. B. W.; Libouban, M. A. A.; Sterrenburg, J. G.; de Wit, J. J. P.; de Vetter, J. R. F.; de Roos, J.; Buijsman, R. C.; et al. (2017) Target Residence Time-Guided Optimization on TTK Kinase Results in Inhibitors with Potent Anti-Proliferative Activity. *J. Mol. Biol.* 429, 2211−2230. <https://doi.org/10.1016/j.jmb.2017.05.014>

[6] Tannous, B.A., Kerami, M., van der Stoop, P.M., Kwiatkowski, N., Wang, J., Zhou, W., Kessler, A.F., Lewandrowski, G., Hiddingh, L., Sol, N., et al. (2013). Effects of the Selective MPS1 Inhibitor MPS1-IN-3 on Glioblastoma Sensitivity to Antimitotic Drugs. *J. Natl. Cancer Inst.* 105, 1322–1331. <https://doi.org/10.1093/jnci/djt168>

[7] Singh, V., Bhoir, S., Chikhale, R.V., Hussain, J., Dwyer, D., Bryce, R.A., Kirubakaran, S., and Benedetti, A.D. (2020). Generation of Phenothiazine with Potent Anti-TLK1 Activity for Prostate Cancer Therapy. *iScience* 23, 101474. <https://doi.org/10.1016/j.isci.2020.101474>
